# OsFKBP12 transduces the sucrose signal from OsNIN8 to the OsTOR pathway in a loosely binding manner for cell division

**DOI:** 10.1016/j.isci.2024.111555

**Published:** 2024-12-09

**Authors:** Zizhang Wang, Hao Li, Yuxiang Weng

**Affiliations:** 1 Key Laboratory of Plant Molecular Physiology, Institute of Botany, Chinese Academy of Sciences, Beijing 100093, China; 2 Laboratory of Soft Matter Physics, Institute of Physics, Chinese Academy of Sciences, Beijing 100190, China

**Keywords:** Molecular biology, Physiology, Plant biology

## Abstract

Previously, OsNIN8 initiated a sucrose signal for cell division in radicle and seed development in rice. Here, a set of genes was induced in starved sprouts after sucrose treatment, and 14 genes were screened between ZH11 and *nin8* as reporters of sucrose signal. Expressions of reporter depended on levels of *OsNIN8* in overexpression and RNAi lines. Further, OsNIN8 interacted with OsFKBP12, a regulator of TOR signal for cell division, and OsFKBP12 interacted with OsTOR (OsTORKD). However, interactions of OsFKBP12 with OsNIN8 or OsTORKD were a loose binding depending on the hydrophobicity of OsFKBP12 C-terminus in Y2H. In addition, OsFKBP12 associating with OsNIN8 was endothermic but with OsNIN8m was exothermic. Knockout OsFKBP12 reappeared *nin8* phenotypes and the complementation of the knockout with C-termini of OsFKBP12 worsened the phenotypes. Treatment with TOR inhibitors caused short radicle and OsTOR RNAi repeated low seed-setting of the phenotypes. So, OsFKBP12 transduced sucrose signal from OsNIN8 to the TOR pathway.

## Highlights

• Sucrose signal induces gene expression
• OsFKBP12 transduces sucrose signal from OsNIN8 to OsTOR via a loose interaction
• The signaling can be blocked separately at OsNIN8, OsFKBP12 or OsTOR proteins
• The target effect of the sucrose signal is cell division

## Graphical abstract

**Figure:**
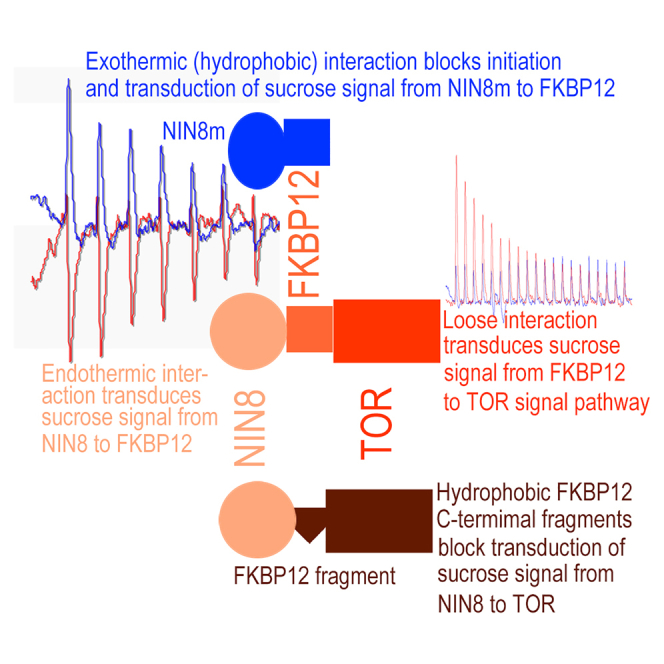

## Introduction

Previously, we found that the mutation of a glycine residue to arginine residue, which increased the charge and the hydrophobicity, in OsNIN8m arrested sucrose signal for cell division in radicle of rice, where the mutation induced coils in secondary structure, lost the second domain, raised the melting temperature for 4°C, folded more tryptophan residues for less autofluorescence, resulting in a compacter enzyme as compared to the wildtype OsNIN8. OsNIN8m bound sucrose lasts for a longer time in an endothermic manner due to the tight interaction but OsNIN8 in an exothermic manner from a loose interaction with sucrose. In addition to this loose structure of interaction for cell division signal, OsNIN8 hydrolyzed sucrose for nutrients and energy metabolism.^1^ We were interested in the signaling depending upon this loose fashion of associations.

It is well known that signal roles of glucose, an alternative carbon source in cells, include glucose repression and glucose promotion in *Saccharomyces cerevisiae* adapted to the availability for fermentation.^2^^,^^3^ In glucose repression, hexokinase 2 (Hxk2) stabilizes the repressor Mig1 binding to the promoter of target genes and recruits protein kinases Snf1, a homolog of mammalian AMP-activated kinase, to the promoter forming a repressor complex to repress gene expression for alternatively metabolic pathways.^4^^,^^5^^,^^6^ Similarly, the accumulation of glucose also represses photosynthesis in mesophyll cells, where Hxk1 forms a complex with H^+^-ATPase B1 and AAA-ATPase subunits of proteasome repressing photosynthesis gene in the leaf of *Arabidopsis thaliana*.^7^^,^^8^^,^^9^

In glucose promotion, two transporter receptors, Snf3 and Rgt2, sense both intracellular and extracellular glucose with an inward-facing and/or occluded conformations in *S*. *cerevisiae*.^10^^,^^11^ The signaling de-represses the repressor Rgt1 for expression of glucose transporter genes, where the receptors adopt glucose for transport.^12^

Obviously, these signals of glucose were only involved in glucose metabolism and transport but not in cell division.

Interestingly, it was photosynthesis but not Hxk1 controlled target of rapamycin (TOR) signaling to activate the cell cycle in root meristems in *Arabidopsis*.^13^ It indicated that TOR signal pathway mediated signals for cell division after sensing carbon source from photosynthesis. This observation was consistent with that the arrest growth of radicle was released with photosynthesis after the age of 3 leaves in our rice mutant *nin8*.^1^

A sucrose-trehalose 6-phosphate (Tre6P) nexus model suggested that Tre6P regulated sucrose levels by sucrose synthesis or consumption, and two protein kinases, SUC-NON-FERMENTING-1-RELATED KINASE1 (SnRK1) and TOR responded to sucrose levels to regulate cell proliferation and meristem size via crosstalk with ABA signaling.^14^^,^^15^

In both cases, it hinted that the TOR pathway is involved in the sucrose signal for cell division in plants. In fact, in mammalian and yeast, it is well known that the mTOR signaling pathway senses nutrients to regulate cell growth^16^^,^^17^ and the 12 kD FK506-binding protein (FKBP12) mediated by rapamycin inhibits the mTOR pathway to arrest cell division,^18^^,^^19^ but gap between nutrients and TOR pathway remains unsolved.

In this work, sucrose as a source of nutrition from endosperm was sensed by OsNIN8 for cell division during germination. The sucrose signal was transduced via OsFKBP12 to activate the OsTOR pathway for cell division in the radicle. OsFKBP12 bound to both OsNIN8 and OsTOR using its hydrophobic C-terminal region forming complexes but associated loosely with both receptors using its hydropathic N-terminal region for signal transduction.

## Results

### Sucrose intensely induces gene expressions and many signal pathways are induced before carbohydrate catabolism

Previously, we found that sucrose but not its metabolites, such as glucose, associating with OsNIN8 initiated signaling for cell division during germination in rice.^1^ To search genes induced by sucrose stimulation, sprouts of ZH11 (WT, 4 days old) were removed from the endosperm and performed fasting for 5 days to run out of sugars, and treated with 175 mM sucrose for 1 h or 6 h. The strategy of comparison of WT with mutant sprouts was not appropriate here because radicles of *nin8* were few but those of ZH11 were long at this time.

**Figure 1:**
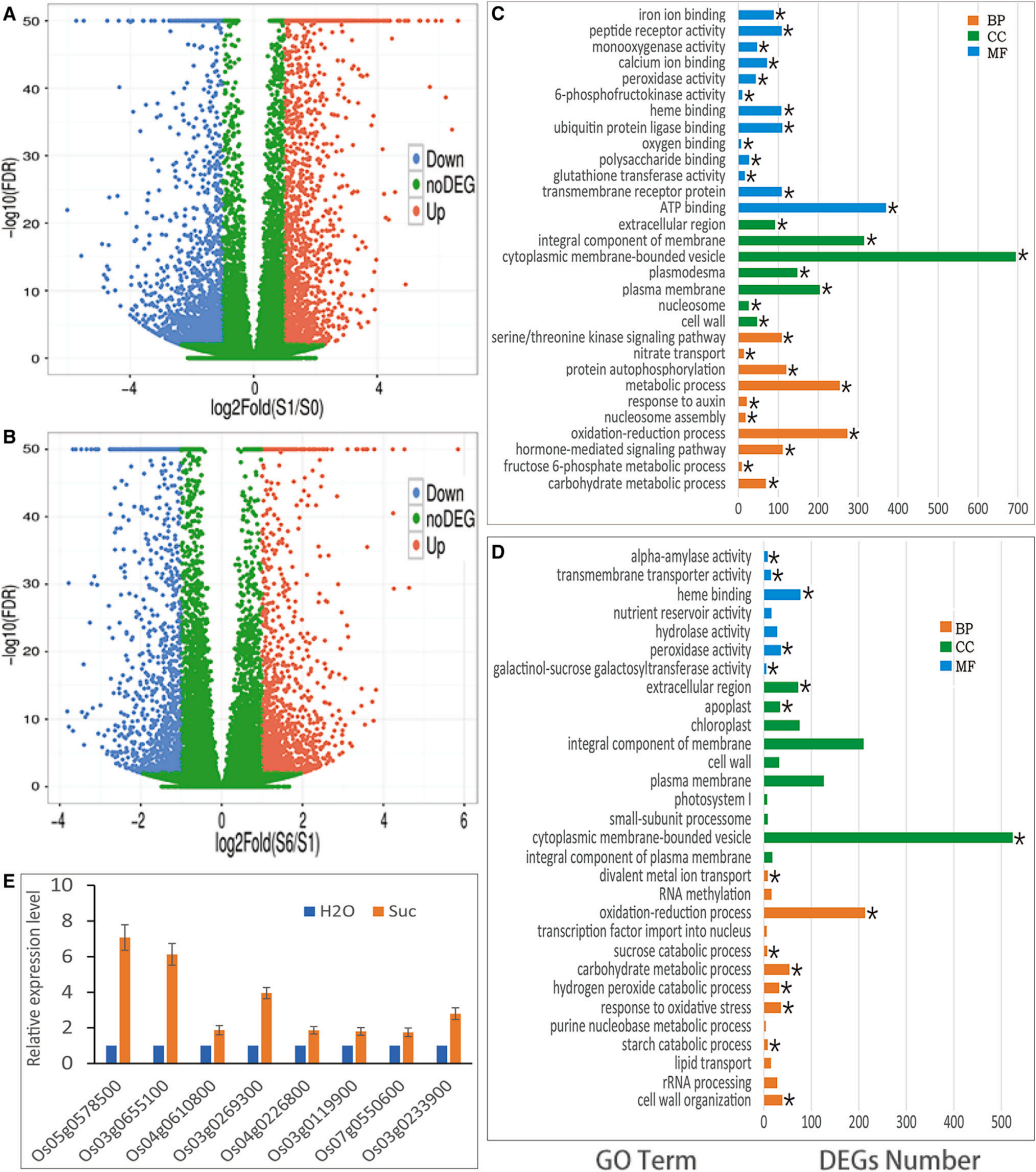
Sucrose inducing gene expressions in sprouts of rice Sprouts of ZH11 were cut from endosperm and starved for running out of sugars, and treated with sucrose for 1 h or 6 h Expression profiling analysis was conducted using RNA-seq, and differentially expressed genes (DEGs) and enrichment were assayed. Experiment was designed with three biological replicates (A and B) Volcano plots of numbers and distributions of DEGS. Showing up-regulation DEGs (red dots) or down-regulation DEGs (blue dots) after sucrose stimulation for 1 h (S1/S0, A) or from 1 h to 6 h (S6/S1, B). Calculation of gene expression using FPKM method, horizontal ordinate indicated differential fold, ordinate indicated q-value. (C and D) Top 30 GO Terms (pathways) of enrichment analyses on DEGs. DEGs number in top GO Terms after sucrose stimulation for 1 h (C) or from 1 to 6 h (D) were listed. GO Term consists of biological process (BP, red), cellular component (CC, green), and molecular function (MF, blue) classes. ∗ indicated significant enrichment (*p*-value <0.05). (E) Confirmation of DEGs by real-time PCR. Some up-regulation DEGs from different GO Terms were confirmed by real-time PCR in sprouts treated with sucrose. Expression levels of control treated with H_2_O were normalized to 1 as a reference. Data were from 3 independent experiments and represented as mean ± SEM. See also Table S1.

Genes of transcriptional responses to sucrose were identified using RNA-seq to compare differential expressions between 0 h vs. 1 h, 1 h vs. 6 h, and 0 h vs. 6 h in three biological repeats following the default filter of the system (Table S1A). There were 3,637 differentially expressed genes (DEGs) including 1,929 up-regulation and 1,708 down-regulation genes after treatment for 1h (S0vsS1) (Figure 1A; Tables 1 and S1B); but it was only 2,539 DEGs (1,316 up and 1,223 down) during subsequent 5 h (S1vsS6) (Figure 1B, Tables 1 and S1C); in total, there were 4,915 DEGs (2,243 up and 2672 down) of S0vsS6 (Tables 1 and S1D). The up-regulation DEGs were 12.9% more than down-regulation DEGs in S0vsS1 (Figures 1A and Table 1), and it was only 7.6% in S1vsS6 (Figure 1B and Table 1); but, conversely, the down-regulation DEGs were 19.13% more than the up-regulation DEGs in S0vsS6 (Figures 1A, 1B, and Table 1).

**Table 1:** Numbers of DEGs in differential expression or enrichment analyses in RNA_seq

	Up^a^	Down^a^	Total	BP^b^	CC^b^	MF^b^	Total
S0vsS1	1929	1708	3637	998	1527	1118	3643
S1vsS6	1316	1223	2539	466	1106	185	1757
S0vsS6	2243	2672	4915	1234	1568	504	3306

a Up and Down regulations of differential expression genes.

b Enrichment analysis of GO Terms (pathways), which consisted of biological process (BP), cellular component (CC), and molecular function (MF) classes. Starved sprouts of ZH11 were treated with sucrose for 1 h and 6 h. Treatment for 1h (S0vsS1), from 1 h to 6 h (S1vsS6), and from 0 h to 6 h (S0vsS6) were analyzed. See also Table S1.

Thus, the inducing effect was more intense, and more genes exhibited up-regulation within the first hour of the treatment, then the effect was weakened.

These DEGs were conducted enrichment analysis following the GO category into GO Terms, which consists of three classes: biological process (BP), cellular component (CC), and molecular function (MF) (Tables S1E–S1G). The 30 most significant enriched GO Terms were listed and a number of significant enriched DEGs in the Term was exhibited (Figures 1C and 1D; Tables S1H–S1J). Similar to differential expression, more enriched DEGs were gathered to top enriched GO Terms in S0vsS1 (3,643 DEGs), it was only 1,757 DEGs in S1vsS6 and 3,306 in S0vsS6. Interestingly, the percentage of enriched DEGs in the MF class in S0vsS1 (30.7%) was more than that in S1vsS6 (10.5%) (Table 1), in which genes are involved in transcription regulator activity in this class.^20^

It was surprising that 36% (361 of 998 genes) of enriched DEGs in BP class involved in GO Terms of signal pathways including transmembrane receptor protein serine/threonine kinase signaling pathway, hormone-mediated signaling pathway, response to auxin and protein autophosphorylation in S0vsS1 (Figure 1C and Table 1). These pathways did not maintain their enrichment in S1vsS6 (Figure 1D and Table 1). However, pathways involved in starch and sucrose catabolism, including GO Terms of starch catabolic, carbohydrate metabolic and sucrose catabolic processes, alpha-amylase and galactinol-sucrose galactosyltransferase activities, gathered in S1vsS6 (Figure 1D and Table 1). Therefore, signal pathways and sugar metabolisms were most enriched inducing in this treatment, and a signal network was induced before starch and sucrose catabolism.

Some DEGs were randomly selected from different GO Terms to confirm their responses to sucrose by using real-time PCR assay. Following the preparation of sucrose treatment for RNA-seq, these sprouts were performed RNA extraction and reverse transcription for Q-PCR. These up-regulation DEGs all enhanced expression in starved sprouts treated with sucrose (Figure 1E).

### Some genes depended on OsNIN8 activity may serve as markers of sucrose signaling

**Figure 2:**
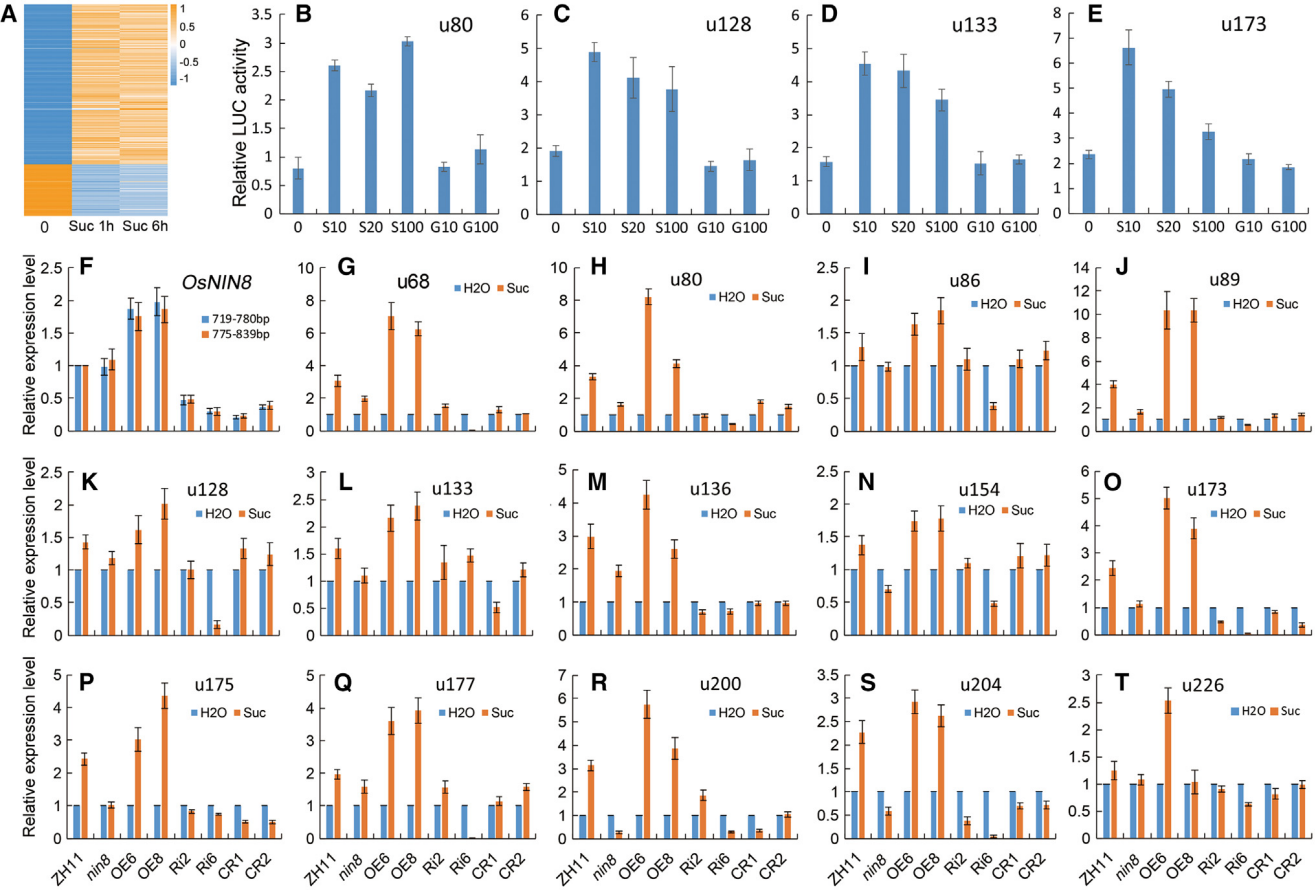
Reporters responding to sucrose are dependent on activity and levels of *OsNIN8* (A) Selection DEGs for screening reporters of response to sucrose from RNA-seq DEGs. The top fold-change 253 up-regulation and 82 down-regulation DEGs, which are differentially expressed after sucrose treatment in ZH11 from the RNA-seq analysis, were selected for screening report genes of response to sucrose using real-time PCR. Finally, 14 genes were well-confirmed differences in response to sucrose in ZH11 against *nin8* from nine independent experiments, which indicated that they depended on the activity of OsNIN8. (B–E) Induced of these reporters responding to sucrose but not to glucose. Promoter of 4 report genes was cloned and constructed into vectors to drive luciferase gene, and these vectors were transformed into rice protoplast. These protoplasts were induced with sucrose or glucose and extracted protein samples for luciferase activities using a luciferase system. S, sucrose; G, glucose, Sugar concentration, mM. Data were from 3 independent experiments and represented as mean ± SEM. (F) Expression of *OsNIN8* was genetically regulated. Expression levels of *OsNIN8* in ZH11, *nin8* (*OsNIN8m*), *OsNIN8* overexpression (OE6 and OE8), *OsNIN8* RNAi (Ri2 and Ri6) and *OsNIN8* knockout (CR1 and CR2) lines in sprouts using real-time PCR were showed. Expression levels in ZH11 were normalized to 1 as control, and two regions of the *OsNIN8* gene as indicated were assayed. Data were from 3 independent experiments and represented as mean ± SEM. (G–T) Expression of report genes was dependent on activity and levels of *OsNIN8*. Expression levels of 14 reporters in starved sprouts of ZH11, *nin8*, OE6, OE8, Ri2, Ri6, CR1, and CR2 lines treated with sucrose were assayed using real-time PCR. Treatment with H_2_0 was as a negative control and the expression level of these controls was normalized to 1 as a reference. Data were from 3 independent experiments and represented as mean ± SEM. See also Table S2 and Figure S1.

Signal pathways have their own downstream responses but there have no marker genes for sucrose signaling so far.^21^ For screening genes as reported, that required sufficiently different responses in this sucrose signaling to meet the low repeatability of the real-time PCR assay, top log_2_Fold 335 DEGs of S0vsS1 including 253 up-regulation and 82 down-regulation genes were selected as candidate genes (Figure 2A; Tables S2A and S2B).

Simultaneously, to find out genes of no-responsive to sucrose in *nin8*, where the sucrose signaling was lost, these candidate genes were screened by differential analysis between ZH11 and *nin8* after treatment with sucrose for 1 h in starved sprouts by real-time PCR. Candidate genes were abandoned gradually when a negative result appeared and 19 candidate genes were saved after three rounds of screening.

These genes were verified for the difference between ZH11 and *nin8* in 9 independent experiments with 3 biological replicates each. Finally, 14 genes, all up-regulation, were confirmed as stably differential responses in ZH11 but not in *nin8* from a criterion of greater than 6 positive results and less than 3 negative results (6+3-) (Table 2). They were named according to their selection number as: u68, u80, u86, u89, u128, u133, u136, u154, u173, u175, u177, u200, u204 and u226.

**Table 2:** Genes identified as sucrose reporters

NO.	Gene ID	Protein	Control	Suc/1h	Fold^a^	Suc/6h	Fold^a^	Pos.^b^	Neg.^b^
u68	Os03g0233900	Non-symbiotic hemoglobin 1	30 ± 0.9	283.4 ± 10.8	9.4	124.2 ± 2.0	4.1	7	2
u80	Os03g0341000	Ethylene-responsive TF RAP2-3	19.8 ± 0.6	150 ± 12.2	7.6	189 ± 12.7	9.5	9	0
u86	Os03g0733800	ER oxidoreductin 1 family protein	91 ± 3.2	368.4 ± 7.3	4.0	248.8 ± 8.3	3.1	6	3
u89	Os03g0816700	Protein LURP-one-related 15	3.5 ± 0.7	47.6 ± 2.3	13.6	40.4 ± 2.7	11.5	8	1
u128	Os05g0524400	ATP-dependent 6-phosphofructokinase	88.3 ± 4.9	646.3 ± 13.1	7.3	626.4 ± 14.3	7.1	9	0
u133	Os05g0572700	Protein phosphatase 2C 51	1.8 ± 0.6	14.4 ± 0.9	8.0	13.8 ± 1.7	7.7	9	0
u136	Os05g0588900	AAA-ATPase	1.2 ± 0.6	8.6 ± 0.17	7.2	11.8 ± 0.38	9.8	8	1
u154	Os06g0548200	Flavin-dependent oxidoreductase	42 ± 1.1	189.2 ± 4.8	4.5	218.5 ± 7.0	5.2	8	1
u173	Os07g0604600	Uncharacterized	360.6 ± 21.1	1846.6 ± 82.3	5.1	1455.2 ± 46.8	4.0	9	0
u175	Os07g0635500	Cytochrome P450 709B2	40.3 ± 1.2	175.3 ± 4.3	4.3	185.3 ± 1.2	4.6	8	1
u177	Os07g0673900	Uncharacterized	103.6 ± 4.7	736.2 ± 23.7	7.1	315.9 ± 15.8	3.0	8	1
u200	Os08g0545500	AP2 and EREBP domain protein	29.1 ± 3.1	218.1 ± 13.6	7.5	227.7 ± 7.9	7.8	8	1
u204	Os09g0471000	Plant cysteine oxidase 1	15.3 ± 1.1	52 ± 0.6	3.4	51.8 ± 1.2	3.4	7	2
u226	Os10g0527400	Glutathione S-transferase GSTU6	33 ± 1.1	168.4 ± 1	5.1	122.6 ± 4.2	3.7	6	3

a Fold changes of gene expression were from data of RNA_seq in ZH11 treated with sucrose for different times against without treatment.

b Positive times (Pos.) and negative times (Neg.) of real-time PCR verification were via responding in ZH11 but not in *nin8* from 9 independent experiments; greater than 6 Pos. and less than 3 Neg. was as stable response. 14 genes from 335 greatest DEGs including 253 up- and 82 down-regulations DEGs were confirmed as stable changes. See also Table S2.

Therefore, these genes responding to sucrose were much more stable dependent on the activity of OsNIN8

Further, to confirm these responses came from sucrose inducing, we used a luciferase report system. Promoters of four response genes (u80, u128, u133, and u173) were cloned and constructed for driving luciferase gene, and these constructs were transformed into rice protoplast. These protoplasts were treated with sucrose or glucose. Fluorescence intensities were assayed. It exhibited that these promoters responded to sucrose but not to glucose *in vivo* (Figures 2B–2E).

To analyze whether these responses depended on expression levels of *OsNIN8* or not *OsNIN8* was overexpressed, RNAi or knockout transformations in ZH11. As expected, levels of *OsNIN8* in overexpression lines were greater than, and those in RNAi or knockout lines were lower than that in ZH11 by real-time PCR assay (Figure 2F).

Then, starved sprouts of these rice lines (WT, mutant, overexpression, RNAi, and knockout) were subjected to sucrose treatment, and expression levels of the 14 reported genes were analyzed by real-time PCR. Again, these genes were highly induced in overexpression lines but repressed in RNAi and knockout lines as compared to in WT or *nin8* (Figures 2G–2T). So, the response of these genes to sucrose depended not only on OsNIN8 activity but also on expression levels of OsNIN8. So, OsNIN8 controlled the initiation of sucrose signal as well as regulated levels of targets in dose-dependent effect.

Similarly, the starved sprouts were treated with different sucrose derivatives or saccharides to test their stimulation to these genes. It showed that the responses to sucrose or maltose > sucrose derivative > non-sucrose disaccharide > trisaccharide > monosaccharide from 6 independent experiments for reliability analysis. Glucose and fructose did not show stable inducing effects overall (Figure S1).

### Possible function of reporter genes

These reporters were only a few genes of those in response to sucrose signal; however, they were screened from the top difference in the first hour of sucrose treatment and stable difference between have and have not sucrose signal in sprouts. The function of these genes would represent some pathways closely related to the sucrose signal for germination. To view these pathways, the function of these genes was investigated, though some genes were functionally unknown and many genes were a member of a gene family or superfamily.

Five genes encoded unknown function polypeptides including u80, which was a fragment of ethylene-responsive TF RAP2-3 containing AP2 domain, u89, which was a member of LURP-one-related 15 scramblase family, u173, which was polypeptide with a B12D domain, u175, which was a member of 709B family of cytochrome P450 superfamily,^22^ and u177, which was similar to a hypoxia induced protein conserved region containing protein.

Interestingly, u200 was also an AP2 and EREBP domain protein, similar to DREB 1J, which was not in response to abiotic stresses unlike other members of the family in rice.^23^ Another one was u204, a fragment similar to plant cysteine oxidase 1, which sensed oxygen in plants.^24^

u68 was a non-symbiotic hemoglobin 1, which was involved in nitric oxide scavenging and had a function in signal for pathways of auxin, ethylene, jasmonic acid, salicylic acid, cytokinin, and abscisic acid hormone during germination.^25^^,^^26^

u154 and u226 were enzymes involved in secondary metabolism, u154 was a berberine bridge enzyme-like, which catalyzes the oxidative reaction for alkaloid and cannabinoid biosynthesis, and alcohol oxidation,^27^ while u226 was similar to the Tau class of glutathione transferases family.^28^

u86 was an ER oxidoreductin 1 family protein, it was involved in the formation of protein disulfide bonds in the rough endoplasmic reticulum.^29^

u128 was an ATP-dependent 6-phosphofructokinase, that catalyzed the phosphorylation of D-fructose 6-phosphate to D-fructose 1,6-bisphosphate in glycolysis pathways.^30^

u133 was a protein phosphatase 2C 51, which was a protein serine ⁄threonine phosphatase for the dephosphorylation of protein.^31^

u136 was an AAA-ATPase, that catalyzed the hydrolysis of ATP to ADP and phosphate ions with releasing energy.^32^

It indicated that these pathways were induced as early as the establishment of sucrose signal during germination, or these cellular processes were required for cell division and growth.

### Association of OsFKBP12 with OsNIN8 is in a loose manner

**Figure 3:**
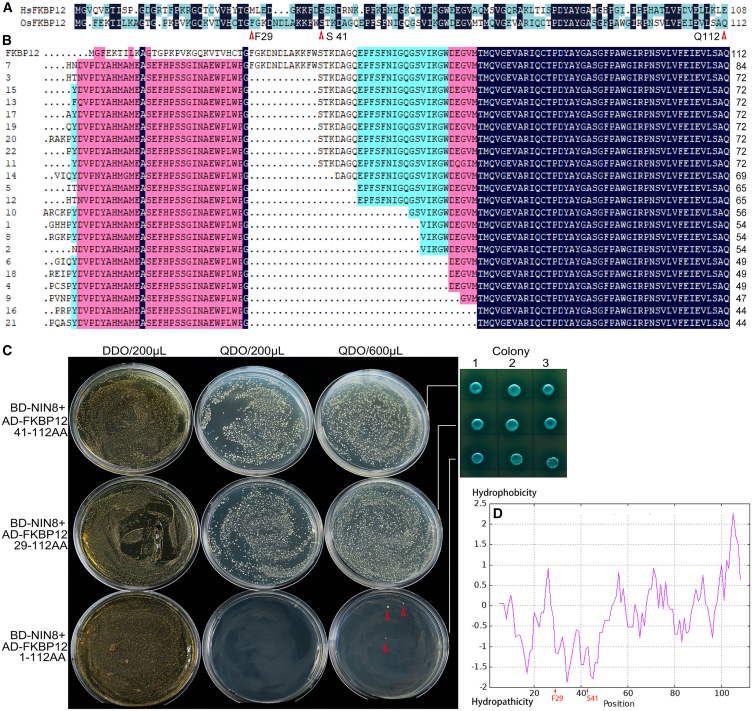
Hydropathicity of the N-terminal region of OsFKBP12 attenuated its interaction with OsNIN8 (A) Alignment of OsFKBP12 with human HsFKBP12a, F29, S41, and Q112 were indicated. (B) 22 fragments of OsFKBP12 screened from a ZH11 rice sprout library in Y2H were aligned with full length OsFKBP12. Sequences before F29 were from the vector of the system and all fragments were C-terminal regions of OsFKBP12 with different lengths. (C) C-terminal of OsFKBP12 interacted well with OsNIN8. Interactions of OsNIN8 with full length (1-112AA), 29–112 AA, and 41–112 AA of OsFKBP12 in Y2H were assayed. Full length OsFKBP12 was unstable interaction with OsNIN8 showing only a few colonies grew on interaction medium (QDO, red arrow) from high inoculation amount (600 μL) and no colony grew from normal inoculation amount; by contrast, its two C-terminal fragments (29–112 AA and 41–112 AA of OsFKBP12) interacted well with OsNIN8, where colonies were in proportion to inoculation amount. 200 μL or 600 μL of double transformed yeast were inoculated as normal or high inoculation amounts, respectively. Blue colonies confirmed the interaction. DDO, double dropout (SD-Leu-Trp) for growth medium; QDO, quadruple dropout (SD-Leu-Trp-His-Ade) for interaction medium. (D) OsFKBP12 was hydropathicity in N-terminus and hydrophobicity in C-terminus. OsFKBP12 was submitted to ProtScale website for hydrophobicity analysis. >0, Hydrophobicity; <0, Hydropathicity. It showed that the N-terminal half was high in hydropathicity with three peaks, while the C-terminal half was high in hydrophobicity. Positions of F29 and S41 of OsFKBP12 were indicated. See also Figures S2 and S3.

To identify proteins involved in sucrose signaling for cell division OsNIN8 was used as a bait for screening a rice sprout cDNA library utilization of yeast two-hybrid system (Y2H). We obtained 22 clones of OsFKBP12, which was about 1/3 of valid clones. All clones lacked their N-termini and the longest one lacked 28 AA (Figures 3A and 3B). 14-3-3 proteins, which interacted with alkaline/neutral invertase in *A*. *thaliana*^33^ were obtained, too.

OsFKBP12 was only 112 AA in length, which was 42.48% of the identity with human FKBP12a (Figure 3A). To verify the interaction, the full length OsFKBP12 was fused to the activation domain （AD-OsFKBP12）of the system for interaction with BD-OsNIN8, and there was no colony grew on selective media (QDO) when the inoculation amount was the same on the growth media (DDO) (Figure 3C). Interestingly, when the inoculation amount increased to 3-folds, a few colonies appeared on QDO, which could also turn blue on medium added X-α-Gal, indicating an unstable interaction between OsNIN8 and OsFKBP12 (Figure 3C).

However, two C-terminal fragments of OsFKBP12, which were cloned according to the two longest fragments from the screen (F29-Q112 and S41-Q112), associated well with OsNIN8 (Figure 3C).

Further, the verification was repeated for BD-OsNIN8m interaction with these AD-OsFKBP12 fragments, and the pattern of interactions reappeared (Figures S2A–S2C). In addition, colonies of two OsFKBP12 fragments interacting with OsNIN8 appeared normally at 4 days but those with OsNIN8m appeared at 7 days after the co-transformation. So, it also indicated that OsFKBP12 fragments interacting with OsNIN8 were different from that with OsNIN8m, which increased its hydrophobicity, in some ways (Figures S2A–S2C).

To confirm these interactions in yeast, expressions of OsNIN8 or OsNIN8m fused with GAL4 DNA BD, and OsFKBP12 or OsFKBP12 C-terminal fragments fused with GAL4-AD were analyzed by immunoblotting (Figures S3A and S3B). These proteins are expressed in these yeast colonies.

Obviously, the N-terminal region of OsFKBP12 attenuated the interaction with OsNIN8 exhibiting a fashion of unstable association in yeast cells. From the model of interaction in Y2H, unstable interactions should result from a loose binding between two proteins. This unstable pattern of interaction was referred to as loose interaction.

To dissect the sequence properties, OsFKBP12 was submitted to the ProtScale website for hydrophobicity analysis. It displayed that AA1-50 was a region of hydropathicity with three hydropathic peaks and the C-terminus was a region of hydrophobicity (Figure 3D). Coincidentally, F29-Q112 lacked peak 1 and S41-Q112 lacked peaks 1 and 2 of hydropathic peaks in OsFKBP12 (Figure 3D). A decrease in hydropathicity must be an increase in the hydrophobicity of two fragments.

Interestingly, the more deletion of these hydropathic peaks the more colonies of OsFKBP12 fragments were obtained in the Y2H screen, where the F29-Q112 fragment had one colony, S41-Q112 fragment had 8 colonies, and fragments of lacked all three peaks had 13 colonies (Figure 3B). So, OsFKBP12 fragments increasing in hydrophobicity reinforced the associations with OsNIN8.

### Association of OsFKBP12 with OsNIN8 is endothermic but with OsNIN8m is exothermic

**Figure 4:**
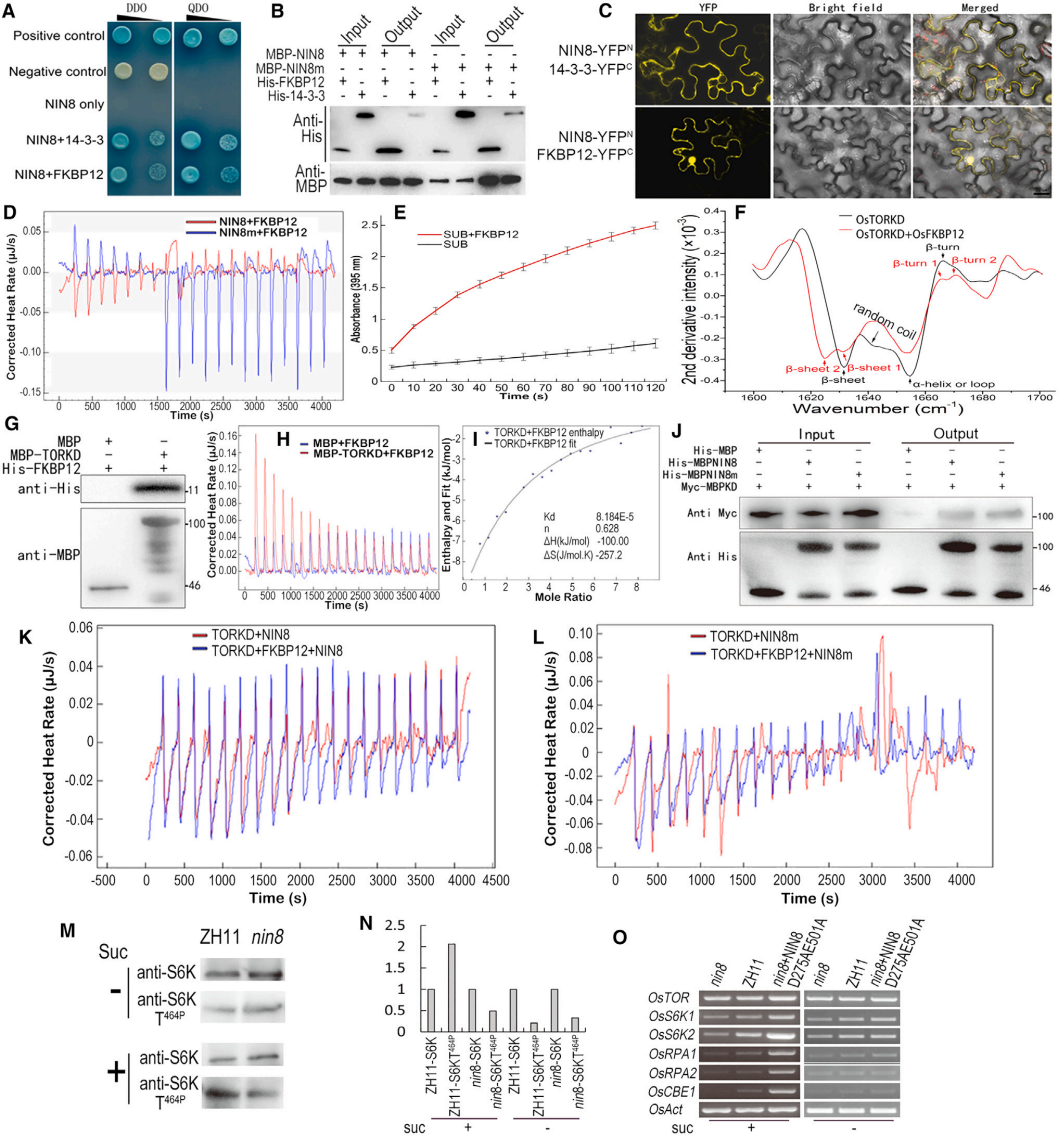
OsFKBP12 interacts with OsNIN8 and OsTOR to transduce TOR signaling (A) OsNIN8 interacted with OsFKBP12 in Y2H. NIN8+FKBP12, BD-OsNIN8 was co-transformated with AD-OsFKBP12; BD-OsNIN8+AD-14-3-3, as an experimental control; single transformation of BD-OsNIN8 for check of self-activation; Negative and positive controls followed the system; DDO, SD-Leu-Trp medium; QDO, SD-Leu-Trp-His-Ade medium; Black triangle above indicated inoculation concentration. (B) OsFKBP12 bound OsNIN8 or OsNIN8m in pull-down assay. Proteins with MBP or His tags were expressed and purified from *E. coli*; Anti-MBP for loading control, anti-His for interaction; 14-3-3, positive control; Negative control saw Figure 4G (MBP+His-OsFKBP12). (C) OsNIN8 interacted with OsFKBP12 in mesophyll cells of tobacco using a BiFC system. 14-3-3, positive control; Negative control saw Figure S4. Yellow fluorescence indicated interaction. Bar = 20 μm. (D) OsFKBP12 bound OsNIN8 or OsNIN8m in ITC assay using OsFKBP12 titrating into OsNIN8 or OsNIN8m. Peak heights getting lower and lower, and then reaching an equilibrium indicated their interaction; Heat rate>0, exothermic; Heat rate<0, endothermic; Inversion of exothermic to endothermic in NIN8m+FKBP12 indicated that the binding reached an equilibrium at injection 8 and a net endothermic association among surplus OsFKBP12; Low exotherm after equilibrium in NIN8+FKBP12 showed the background peaks of the titration. (E) OsFKBP12 had peptidyl propyl *cis*-*trans* isomerase activity on a polypeptide substrate (SUB). OsFKBP12 was added to the substrate in potassium phosphate buffer and recorded the OD_395_ for 2 min with 20 s intervals. Data were from 3 independent experiments and represented as mean ± SEM. (F) Second structure of OsTORKD changed after reaction with OsFKBP12 using FTIR spectroscopy assay. MBP-OsTORKD was expressed and purified from *E. coli*. Changes of β-sheet (1631 cm^−1^), β-turn (1676 cm^−1^), and random coil (1645 cm^−1^) of OsTORKD after reaction with OsFKBP12 were shown. (G) OsFKBP12 bound OsTORKD in a pull-down assay. MBP+His-OsFKBP12 as a negative control. (H) OsFKBP12 bound OsTORKD in ITC assay using His-OsFKBP12 titrating into MBP-OsTORKD. The characteristic peaks of association (peaks of titration getting lower and lower until titration saturation) appeared. His-OsFKBP12 titrating into MBP-tag as a negative control. (I) Fitting curve of OsFKBP12 interacting with OsTORKD in ITC assay. Mole ratio and binding parameters of the association were indicated. (J) OsTORKD bound OsNIN8 or OsNIN8m in pull-down assay. His-MBP, His-OsNIN8, OsNIN8m, and Myc-OsTORKD were expressed and purified from *E. coli*; Anti-His for loading control, anti-Myc for interaction. MBPKD, MBP-OsTORKD; MBP+MBPKD as negative control. (K and L) OsNIN8 (K) or OsNIN8m (L) bound OsTORKD with or without OsFKBP12 in ITC assay. OsNIN8 or OsNIN8m titrating into OsTORKD with or without the presence of OsFKBP12 showed similar endothermic interactions; titrating peaks consisted of an exothermic background peak and a lagging endothermic peak each, which displayed the endothermic association. OsFKBP12 did not change associations of OsTORKD with OsNIN8 or OsNIN8m shown. (M and N) Phosphorylation of OsS6K1 was enhanced upon sucrose treatment in ZH11 but not in *nin8*. (M) Phosphorylation level of OsS6K1 T^464^, a target of TOR signaling, in ZH11, enhanced after sucrose treatment but that was not in *nin8*. Protoplasts were transiently transformed with S6K1 overexpression construct and treated with sucrose to prepare protein samples. Anti-S6K indicated loadings of S6K1 protein and anti-S6KT^464p^ indicated phosphorylation level of OsS6K1 T^464^. Anti-S6KT^464p^ antibody was blocked with antigen polypeptide of Anti-S6K before use to avoid possible cross contamination. (N) Comparison of blotting bands in digitization. Loading of S6K1 was normalized to 1 and phosphorylation levels of S6KT^464p^ were compared between in ZH11 and in *nin8* with (Suc +) or without (Suc -) sucrose treatments. (O) Transcription of many downstream genes enhanced upon sucrose treatment in ZH11 but not in *nin8*. Transcriptional levels of *OsTOR*, *OsS6K1*, *OsS6K2*, *OsRPA1*, *OsRPA2,* and *OsCBE1* in ZH11, *nin8,* and catalytic mutation line (*nin8*+NIN8D275AE501A) were determined using RT-PCR, *OsAct* as internal standard; Control experiment of without sucrose treatment was as negative control. See also Figures S3–S7 and Table S3.

The interaction of OsFKBP12 with OsNIN8 in Y2H (Figure 4A) was confirmed by pulldown assay *in vitro*, in which both OsNIN8 and OsNIN8m could interact with the full length of OsFKBP12, those with Os14-3-3 served as the positive control (Figure 4B).

To confirm these interactions *in vivo*, a BiFC assay was performed as described^34^ in tobacco leaves. Both OsNIN8 with Os14-3-3 or with OsFKBP12 could reproduce fluorescence of interactions but OsNIN8-Os14-3-3 complex localized in cytoplasm and OsNIN8-OsFKBP12 complex localized in cytoplasm as well as in cell nucleus (Figure 4C). The negative controls did not show interaction (Figure S4).

To explore interactions of OsNIN8 or OsNIN8m with OsFKBP12 thermodynamically, OsFKBP12 was titrated into OsNIN8 or OsNIN8m for enthalpy change analysis involving the use of isothermal titration calorimetry (ITC) assay. The getting lower and lower pattern of peak heights displayed their bindings. Surprisingly, OsNIN8 binding OsFKBP12 was endothermic but OsNIN8m binding OsFKBP12 was exothermic (Figure 4D). Their bindings reached an equilibrium at injection 8 (Figure 4D). Therefore, the charge and hydrophobic increase in OsNIN8m reversed its thermodynamic association with OsFKBP12.

### Loose association of OsFKBP12 with OsTORKD

FKBP12 is a peptidyl-prolyl *cis*-*trans* isomerase acting as a molecular chaperone in facilitating protein folding.^35^ The important function of FKBP12 was its interaction with TOR, a Ser/Thr kinase, forming a complex to serve as the central controller of the TOR signal pathway for the G1-S transition of cell cycle sensing available nutrients.^36^^,^^37^^,^^38^ Both OsFKBP12 and OsTOR homologs were single genes in the rice genome.^39^^,^^40^

To test the isomerase activity of OsFKBP12, the isomerization of a proline residue in a peptide substrate was measured following the method described.^35^ After adding OsFKBP12 the reaction of isomerization was boosting and continuous (Figure 4E). Thus, OsFKBP12 conserved its evolutionary function in rice.

TOR was large and its structure included a kinase domain (KD) (Figure S5A), which was always investigated for its function.^41^^,^^42^^,^^43^^,^^44^^,^^45^ KD of OsTOR (OsTORKD) was cloned according to the conservation of TOR structure.^41^ This fragment was fused to MBP and expressed in *E*. *coli*.

To have an insight into changes in the secondary structure of OsTORKD after reaction with OsFKBP12, OsTORKD, and OsTORKD+OsFKBP12 were compared using FTIR analysis. The β-sheet at 1631 cm^−1^, the β-turn at 1676 cm^−1^, and the random coil at 1645 cm^−1^ of OsTORKD were changed (Figure 4F). OsFKBP12 altered the secondary structure of OsTORKD suggesting their interaction without rapamycin.

*Arabidopsis* FKBP12 interacted with FKBP12-rapamycin binding domain (FRB), which harbored in KD, of TOR (Figure S5A) and regulated TOR signal mediated by rapamycin in plant cells.^42^ To confirm the interaction of OsTORKD with OsFKBP12, MBP-OsTORKD was performed pulldown assay with His-OsFKBP12 and it displayed their interaction *in vitro* (Figure 4G). Further, this interaction was confirmed by ITC and it showed a stoichiometric affinity (Figures 4H and 4I) with a mole ratio between OsTORKD and OsFKBP12 of 1:6 (Figure 4I), in addition to the pattern of interaction (Figure 4H). Again, OsTOR could be associated with OsFKBP12 without being mediated by rapamycin.

To dissect the interaction in Y2H, which did not occur between AtFKBP12 and AtTORKD,^43^^,^^44^^,^^45^ we followed the loose association of OsFKBP12 with OsNIN8 (Figures 3 and S2). Combining the full-length, F29-Q112, and S41-Q112 of OsFKBP12 fragments with the KD (H1896-W2465), FRB domain (H1896-T2014), and KD without FRB (KD-FRB, T2015-W2465) of OsTOR fragments, respectively, were performed in Y2H (Figures S5A and 5SB). The full-length OsFKBP12 with FRB of OsTOR and the F29-Q112 of OsFKBP12 with KD of OsTOR reproduced the loose associations (only a few colonies appeared), both F29-Q112 and S41-Q112 of OsFKBP12 with FRB of OsTOR were strong interactions (Figures S5B and S5C). KD-FRB (without FRB) served as the negative control. Again, the interaction of OsTOR with OsFKBP12 was in the manner of loose association depending on the hydrophobicity of OsFKBP12.

To confirm these interactions in yeast, expressions of OsTORKD, OsTORKD without FRB or OsTORFRB fused with GAL4 DNA BD, and OsFKBP12 or OsFKBP12 C-terminal fragments fused with GAL4-AD were analyzed by immunoblotting (Figures S3A and S3B). These proteins are expressed in these yeast colonies.

To estimate interactions of OsTORKD with OsNIN8 or OsNIN8m, these interactions were confirmed in a pulldown assay (Figure 4J). Further, OsTORKD with or without OsFKBP12 were titrated with OsNIN8 or OsNIN8m, respectively, using ITC. Their peak shapes were similar and showed two steps, an exothermic peak, which was about the same height indicating the background of titration, and then an endothermic peak, which was getting lower and lower indicating their interactions. So, both bindings of OsTORKD with OsNIN8 or with OsNIN8m were endothermic except that OsNIN8m interacting with OsTORKD magnified its peak heights after reaching equilibrium (Figures 4K and 4L). Interestingly, OsFKBP12 did not alter the binding patterns of OsTORKD with OsNIN8 or with OsNIN8m showing that the binding of OsTORKD with OsFKBP12 was independent of the binding of OsNIN8 with OsFKBP12 (Figures 4K and 4L).

It demonstrated that OsNIN8, OsFKBP12, and OsTORKD were interacting with one another forming a ternary complex, and that OsFKBP12, as a molecular chaperone for protein folding, interacted loosely with both OsNIN8 and OsTOR at least with 6 OsFKBP12 molecules for a complex.

### Activation of the TOR signal pathway depends on the activity of OsNIN8 and sucrose stimulation

To investigate whether OsNIN8 controls the TOR signal pathway or not, we tested the response of TOR downstream targets depending on the activity of OsNIN8. Phosphorylation of the translation regulators ribosomal S6 Kinase 1 (S6K1) and eukaryotic translation initiation factor 4E binding protein 1 (4E-BP1) were the two best-known targets of TOR for ribosome biogenesis and translation initiation.^38^^,^^46^^,^^47^

To detect the phosphorylation of OsS6K1 T^464^, which corresponded to T^389^ of human S6K1 and T^449^ of AtS6K1,^42^ a 15-AA peptide with or without phosphorylation modification at T^464^ were synthesized for the preparation and purifying of polyclonal phosphorylation and non-phosphorylation antibodies of OsS6K1. Antibodies were prepared by Abcam (Shanghai) and met the specificity (Figure S6).

Following the cell assay for S6K1 phosphorylated by endogenous TOR,^42^ protoplasts of ZH11 and *nin8* were transiently transformed with *OsS6K1* expression vector and treated with or without sucrose to prepare protein samples. Immunoblots with non-phosphorylation antibodies were for OsS6K1 expression and with phosphorylation antibody for OsS6K1 T^464^ phosphorylation level. Levels of S6K protein or phosphorylation were compared against loading controls. It was only in ZH11 where the level of OsS6K1 T^464^ phosphorylation was augmented under sucrose treatment but not augmented without sucrose treatment, comparatively, the phosphorylation levels were not augmented regardless of sucrose treatment in *nin8* (Figure 4M).

Blotting bands were quantified by area and density and the phosphorylation level in ZH11 was about four times that in *nin8* (Figure 4N). This result indicated that OsS6K1 T^464^ phosphorylation depended on the activity of OsNIN8, in which the TOR signal pathway worked.

To confirm transcriptionally the TOR-S6K/4E-BP1-ribosome biogenesis signaling pathway depended on OsNIN8, expressions of *OsTOR*, *OsS6K1*, *OsS6K2*, *Os4E-BP1* (nominated *CBE1* in rice,^48^) DNA-directed RNA polymerase Ⅰ subunits *OsRPA1* and *OsRPA2* were assayed in sprouts of *nin8*, ZH11, and *nin8*+*NIN8D275AE501A* complementation, which were treated with or without sucrose using RT-PCR (Table S3).

It showed that expressions of *OsTOR* among three lines were just as actin gene, the internal standard, but expression levels of *OsS6K1*, *OsS6K2*, *OsCBE1*, *OsRPA1,* and *OsRPA2* were generally low in *nin8*, and increased in ZH11 after sucrose treatment. Comparatively, the expression of these genes among the three lines was unchanged in sprouts without sucrose treatment as a negative control (Figure 4O).

Interestingly, they were intensely promoted in the *nin8*+*OsNIN8D275AE501A* line or due to an over sucrose treatment but a lack of energy supply.

Therefore, TOR signaling here was stimulated by sucrose mediated by OsNIN8 activity.

### Hydropathically loose association of OsFKBP12 regulates the TOR pathway

**Figure 5:**
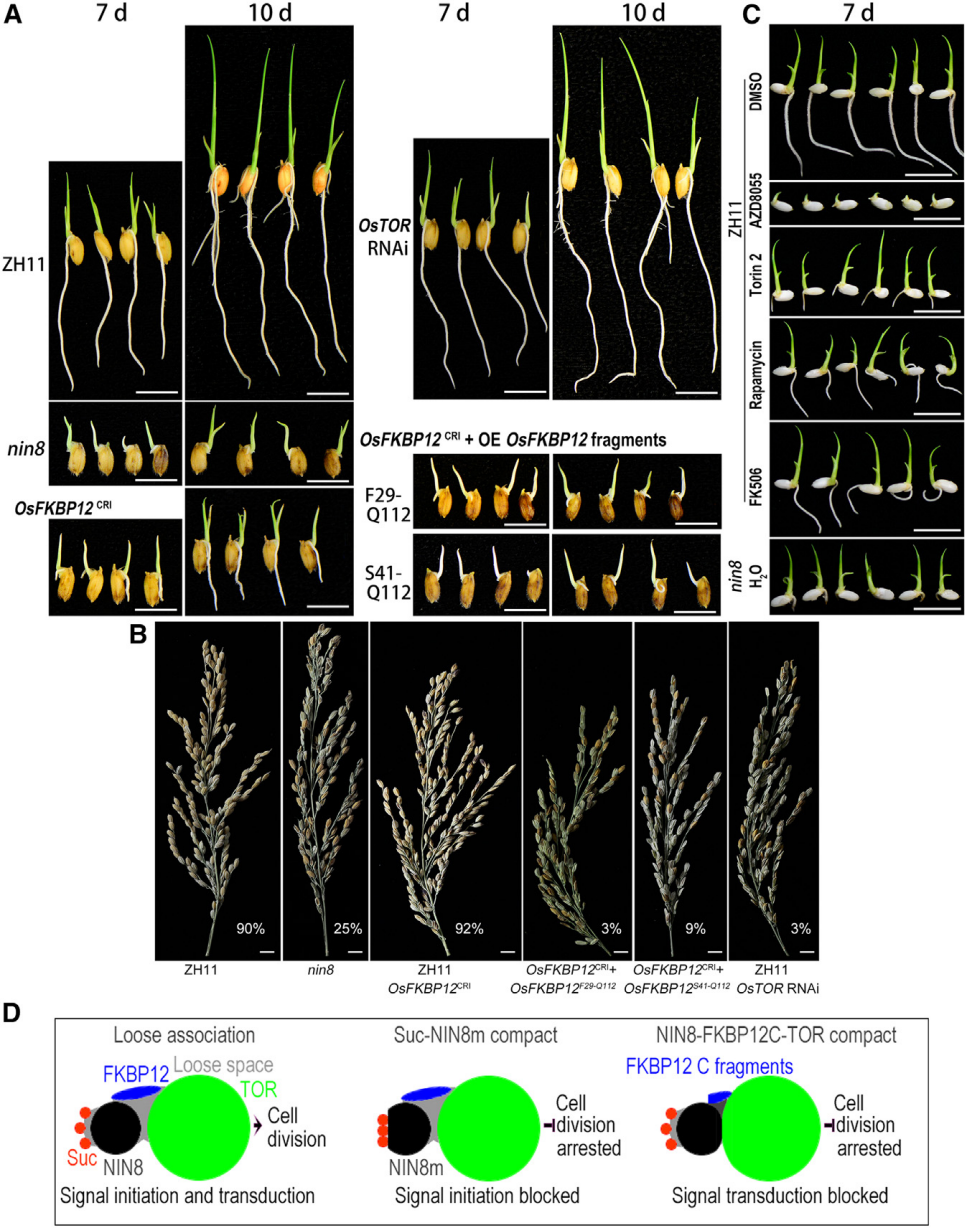
*OsFKBP12* and *OsTOR* independently control the elongation of radicles and seed setting in rice (A) Knockout of *OsFKBP12* reappeared short radicle (*OsFKBP12*^*CRI*^). Its degree was less severe than in *nin8* comparatively. However, *OsFKBP12*^*CRI*^ line complemented with overexpression of F29-Q112 and S41-Q112 fragments of OsFKBP12 encoding regions (*OsFKBP12*^*CRI*^+OE *OsFKBP12* fragments) aggravated the phenotype at DAI 7 and DAI10. Knockout of gene using CRISPR/Cas9; F29-Q112 and S41-Q112, C-terminal fragments of *OsFKBP12*. Phenotypes of ZH11 and *nin8* served as positive and negative controls, respectively. Phenotype of OsTOR RNAi was shown. Bar = 1 cm. (B) Attenuations of *OsNIN8*, *OsFKBP12,* or *OsTOR* all resulted in low seed settings. Various lines of rice are harvested from the same plot. *OsFKBP12*^CRI^, knockout; *OsFKBP12*^CRI^+*OsFKBP12*^F29-Q112^ and *OsFKBP12*^CRI^ +*OsFKBP12*^S41-Q112^, knockout line was overexpressed by two *OsFKBP12* fragments for complementation. Seed set rate was provided alongside. Bar = 1 cm. (C) Treatment with four TOR-specific chemical inhibitors led to short radicle phenotype. Seeds of ZH11 were exposed to 4 inhibitors on media during germination. AZD8055, Torin 2, Rapamycin, and FK506, inhibitors; DMSO, the dissolvent, served as reagent control (high concentration of DMSO itself inhibited germination); *nin8* without treatment served as phenotype control. Bar = 1 cm. (D) Diagram of the loose association among Suc-NIN8-FKBP12-TOR activated TOR signaling and cell division. Compact associations of Suc-NIN8m, NIN8-FKBP12C/FKBP12C-TORKD did not activate TOR signaling and cell division; FKBP12C, C-terminal fragments of OsFKBP12. Loose spaces between or among molecules were indicated in gray. See also Figure S8.

FKBP12 regulates allosterically TOR kinase activity for cell division,^38^ knockout of *OsFKBP12* was conducted using CRISPR/Cas9 technology. *OsFKBP12*^CRI^ knockout line was obtained with a C-insertion after 48-bp and its protein sequence was frameshift after 16 AA and stopped at the 33 AA position. Radicles of *OsFKBP12*^CRI^ were short similar to that of *nin8*, but to a lesser degree of arrest, appearing as short radicles during germination (Figure 5A).

Interestingly, the seed set rate of *OsFKBP12*^CRI^ tended to be normal (Figure 5B). So, deficiency of OsFKBP12 for loose interactions with OsNIN8 and OsTOR impaired but did not completely arrest TOR signaling such as the knockout or mutation of OsNIN8. It might be the case that the OsFKBP12 as a linker of the signaling was opened but not blocked in this line of rice.

Because fragments F29-Q112 and S41-Q112 of OsFKBP12 were strongly associated with OsNIN8 or OsTOR and lost the loose fashion of regulation function (Figures 3C, S2, and S4), two fragments of OsFKBP12 were over-expressed in *OsFKBP12*^CRI^ for complementation. Interestingly, they showed very short in radicles, shorter than *OsFKBP12*^CRI^ closing to the degree of *nin8* (Figure 5A). Their seed set rates were very low, even lower than that of *nin8* (Figure 5B).

Therefore, the hydrophobic binding of the C-termini of OsFKBP12 with OsTOR blocked the TOR signaling in rice. Obviously, the loose association of the N-terminus of OsFKBP12 played a role in transducing the sucrose signal to the TOR pathway.

So, the hydropathic N-terminal of OsFKBP12 allowed a loose association in the ternary complex to regulate the activity of OsTOR. This regulation was consistent with effects of hydrophobically steric hindrance of FKBP12,^49^ incorporation of hydrophobic rapamycin, FK506, and glycine mutation into hydrophobic arginine of OsNIN8m, all these increases of hydrophobicity arrested cell division. Thus, TOR signaling was susceptible to hydrophobic regulation.

Further, hydrophobic and hydropathic properties of FKBP12s from rice, *Arabidopsis*, yeast, and humans were compared. Those from rice and yeast were rich in hydrophobicity but those from *Arabidopsis* and humans were rich in hydropathicity (Figure S7). These differences should lead to no complementation of these FKBP12s associating with TORs.^43^^,^^44^^,^^45^

### Knockdown and inhibition of OsTOR reappear the seed set and radicle phenotypes

Knockout of *OsTOR* could not obtain homozygote of frameshift or code stop mutants following previous embryo lethality.^42^ Interestingly, RNAi of *OsTOR* did not lead to short radicles (Figure 5A) but an extremely low seed set rate (Figure 5B). Reproductive development was more susceptible to *OsTOR* RNAi than radicle growth.

Treatment of TOR-specific chemical inhibitors decreased cell division.^50^ To inhibit the OsTOR pathway two ATP-competitive TOR kinase inhibitors, AZD8055 and Torin2, and two TOR/FKBP12 complex allosteric inhibitors, rapamycin, and FK506, were treated on seeds during germination via adding to medium.

All four inhibitors were hydrophobic and dissolved in DMSO, but the concentration of DMSO in the medium greater than 1% (v/v) would inhibit the growth of sprouts in rice. After the concentration gradient experiment, exposure concentrations of AZD8055, Torin2, rapamycin, and FK506 were 300, 85, 480, and 45 μM in media, respectively, in this treatment.

Four inhibitors could inhibit the growth of radicles of ZH11 to different degrees, showing short radicles but leaf emergence rate unchanged except for the inhibition by AZD8055, which was due to an over inhibition on the growth of both radicles and leaves at this concentration (Figure 5C).

Phenotypes of short radicle but the speed of producing leaves unchanged were similar to those in *nin8*, *OsFKBP12*^CRI^ and *OsFKBP12*^CRI^ + *OsFKBP12*^CRI^ fragments lines (Figure 5A) and these phenotypes were characteristics of TOR pathway specific inhibition.^50^

So, the attenuations of the TOR pathway by *nin8*, *OsNIN8*^CRI^s, *OsFKBP12*^CRI^s, OsTOR RNAi, and treatment of TOR inhibitors shared similar phenotypes in spite of action at different links of the pathway. Thus, OsNIN8, OsFKBP12, and OsTOR controlled the TOR signaling pathway independently in this pathway.

**Figure 6:**
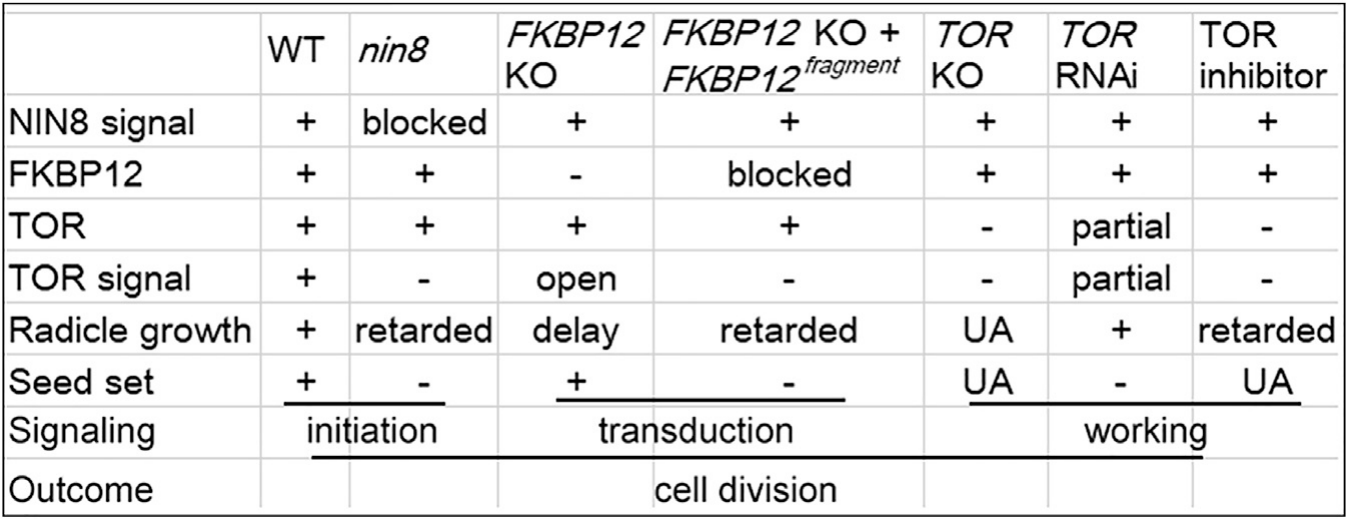
Genotype and phenotype of genetic modification lines and their roles within the TOR pathway in rice *FKBP12* KO + *FKBP12*^fragment^, complementation of OsFKBP12 with *F29-Q112* or *S41-Q112* fragments of *OsFKBP12;* KO, knockout; UA, unavailable.

Since OsNIN8, OsFKBP12, and OsTOR could interact with one another (Figure 4) as well as OsNIN8 bind sucrose in a similar loose manner (Figures 3, S2, and S5, Wang et al. 2024^1^), that achieved the signaling for cell division, regulation of the signal pathway was either in different domains of the quaternary complex by maintaining the complex in a loose status or step by step for triggering and transduction of the signal along the TOR pathway (Figure 6).

## Discussion

Gene expressions responding to sucrose were not all mediated by sucrose signal pathways, just as in previous observations, the effect of sucrose on germination included nutrition and signal effects,^1^ nutrition met the need for a metabolic network but signaling was involved in some specific pathways. Cell division and elongation would go on when nutrition was present, and stop when nutrition was absent, but cell division would not happen when OsNIN8 was mutated into OsNIN8m even if high accumulation of nutrition because the cell division pathway did not receive signaling from available sucrose.

### Genes in response to sucrose signal during germination are in order expression of pathways for cell division

From the sink-source relationship, the central target of sucrose was for cell division and growth during germination. The mass flow of sucrose for cell division was a convenient model for sucrose effects. Clear removal of endosperms and starvation made cells run out of sugars and other nutrients to minimize influencing factors.

It had been reported that some gene expressions were induced by sucrose. For example, genes for biosynthesis of starch, such as ADP-glucose pyrophosphorylase (AGPase), in the detached leaf of sweet potato^51^ and during the developing endosperm of maize,^52^ were enhanced by sucrose treatment. Similarly, genes for accumulations of anthocyanin^53^ or fructan^54^ were also induced by sucrose in leaves. However, these inductions would not enhance during germination because the direction of metabolic flow reversed those in leaves or developing endosperms, where sucrose was polymerized into starch for deposition.

Ribosome synthesis and cell division were the downstream processes of sucrose metabolism, relative genes would be induced by sucrose during germination. Nucleolin gene, *AtNuc-L1*.^55^ or D-type cyclins, *CycD2* and *CycD3*^56^ were strongly induced by sucrose, but they also could be induced by glucose in *Arabidopsis*, showing non-specific to sucrose.

From Table S1A, there were 71 genes relative to ribonucleoprotein complex appeared from the experiment, but no DEG in S0vsS1 (Table S1B), and 9 DEGs in S1vsS6 (all up-regulation) (Table S1C). Similarly, there were 38 genes relative to cyclin-dependent protein serine/threonine kinases (CDKs) and regulation of CDKs, and only three DEGs (two up- and one down-regulation) in both S0vsS1 and S1vsS6. Expression of ribonucleoproteins was not induced after sucrose treatment for 1 h, but was induced after then. Genes for the cell cycle seemed they had not started at all.

In addition, the three DEGs (u49, u157, and u245) encoded Cyclin-like F box domain containing protein were selected as candidates (Table S2) for screening reporters, but they all could not be confirmed stably.

Comparatively, the results that signal pathways enriched in the first hour and carbohydrate metabolism after then in the treatment, ribosome synthesis for protein synthesis and cell cycle for cell division to restart growth of the starved sprouts would be enhanced later.

### Loose association of OsFKBP12 with OsNIN8 or OsTOR is a weak association in Y2H

In the early days of this Y2H, when interacting was determined by *β*-galactosidase activity, transcriptional activity of the two hybrid proteins consisting of GAL4 BD-SNF1 and SNF4-GAL4 AD was only 4.5% of that of wild type GAL4 protein.^57^ In two hybrids of SIR proteins, interactions of different fragments of SIR4 fused to AD with the same BD fused protein led to different levels of activity.^58^ Mutation of the positive control of this Y2H, p53, into different mutants, which affected its binding to SV40 large T antigen, produced colonies with colors from dark blue, light blue, very light blue to white and corresponding *β*-galactosidase activities from 150, 34–94, 7.3–7.5 to 0.8–2.3 because of reducing affinity.^59^

Recently, the levels of interactional activation of Y2H were developed to measure by NanoLuc luciferase as a quantitative Y2H, which could identify weak interactions that displayed variable growth in the Y2H medium.^60^ In fact, in addition to weak interactions of Y2H attenuated expressions of auxotrophic complementation, the growth of colonies required four independent reporter genes being bound to promoters and activated at the same time. These factors highly restricted colonies' growth on the selection medium.

In the case of OsFKBP12, the weak interaction of the full-length protein with OsNIN8 or OsTORFRB in this Y2H could be evaluated from the parallel assay of the strong interactions of its c-terminal fragments. A great many fragments of OsFKBP12 were screened from the library but not a single of the full-length OsFKBP12 was obtained. Fragments of OsFKBP12 two hybrid with OsNIN8 or FRB of OsTOR presented colonies on medium normally but it was reluctant for the full-length OsFKBP12.

### OsFKBP12 is via the C-terminus for binding and the N-terminus for signal transduction

Like other FKBP12s, OsFKBP12 has also consisted of 6 β-sheets, 2 α-helixes and 3 loops^61^ (Figure S2B). F29-Q112 fragment lacked the β1-β2 and the S41-Q112 fragment lacked β1-β2-40s loop regions, which corresponded to lacking one or two hydropathic peaks, respectively (Figure 3D). The 80s loop harbored close to the C-end of FKBP12s.

It had been structurally determined that FKBP12 associating with receptor partners was mainly via its C-terminal region, in particular the 80s loop, which could interchange conformations^62^ and respond to signaling via a hydrophobic notch within interactions.^63^ For example, FKBP12 associated with TOR,^64^ calcineurin,^65^ type Ⅰ transforming growth factor β receptor^66^ or bone morphogenetic protein receptor ALK2,^67^ all were mediated by the hydrophobic C-terminus.

In rice, two C-terminal fragments associated well but the full-length OsFKBP12 showed the loose fashion association with OsNIN8 or OsTORKD in Y2H. Knockout of OsFKBP12 restricted the growth of root into *nin8* phenotypes, and its complementation with C-terminal fragments aggravated these phenotypes.

So, its C-terminal region enhanced the bindings with receptors using higher hydrophobicity, and the N-terminal region executed signal regulation using higher hydropathicity. As a molecular chaperone,^35^ balance of hydropathicity and hydrophobicity was the regulation fashion of FKBP12 in different contexts.

### OsFKBP12 transduces sucrose signal via favor of thermodynamics for cell division

In mammals, FKBP12 binding to hydrophobic FK506 or rapamycin to inhibit the immune activation of the mTOR pathway as an immunophilin was involved in hydrophobic regulation or steric hindrance of its activity for cell division.^18^^,^^41^^,^^49^^,^^68^^,^^69^^,^^70^ mTOR is a signaling hub coordinating the availability of nutrients for cell growth.^71^ But little was known about the mechanism of signal transductions.

Here, OsFKBP12 is associated with both OsNIN8 and OsTOR, particularly, a 1:6 mol ratio with OsTORKD. The association was in a loose fashion, which hydropathic N-terminus of OsFKBP12 made the association easily. This association allowed TOR activation and signaling for cell division, any damages to it such as the knockout or complementation of OsFKBP12 with its hydrophobic C-termini led to TOR arrest.

Importantly, combining with associations among Suc/OsNIN8 (exo), OsNIN8/OsFKBP12 (endo), and OsFKBP12/OsTOR (exo) conducted sucrose signaling in WT for normal cell division, while with Suc/OsNIN8m (endo), OsNIN8m/OsFKBP12 (exo) and OsFKBP12/OsTOR (exo) arrested the signaling and cell division in *nin8*. These thermodynamic processes were molecular bases of the loose association for the initiation and transduction of sucrose signal.

### Hydropathic modification of OsFKBP12 or is a way in the regulation of TOR signal

The kinase activity of mTORC1 can be inhibited by hydrophobic reagents^19^ and activated allosterically by a small GTPase, Rheb,^72^ Amino acids such as leucine, glutamine, or arginine were sensed by members upstream of mTOR kinase,^73^^,^^74^ but how these signals transmit to mTOR are not known.

Here, OsTOR activity was arrested when OsNIN8 or OsFKBP12 increased in hydrophobicity to strengthen these interactions into some compact states, which included the mutation of OsNIN8 into hydrophobic OsNIN8m, and complementation of OsFKBP12 with its hydropathic reducing fragments. In addition, attenuation of the OsTOR pathway by hydrophobic TOR inhibitors. These operations altered hydropathicity to regulate OsTOR activity. Therefore, hydropathic or hydrophobic degrees of regulatory factors or played an important role in the TOR signal.

### OsTOR signaling can be controlled at different links independently

Knockout of OsNIN8 (*OsNIN8*^CRI^s) could not activate OsTOR because OsNIN8 was a sensor for initiating sucrose signal, hydrophobic mutation of OsNIN8 (OsNIN8m) also could not activate OsTOR because the site of sucrose accommodation on OsNIN8 was compact blocked to extinguish sucrose signal, but overexpression of OsNIN8 enhanced susceptibility of sucrose signal to activate OsTOR pathway.

Knockout of OsFKBP12 (OsFKBP12^CRI^) left OsTOR inactive because it disconnected the sucrose signal to the TOR pathway, complementation of *OsFKBP12*^CRI^ with C-termini of *OsFKBP12* also could not activate OsTOR because the loose fashion of OsFKBP12 stimulating OsTOR was hydrophobic blocked into compact binding.

Finally, the attenuation of OsTOR activity by RNAi or treatment with inhibitors led to OsTOR unstable or being inhibited.

Here, an exothermic OsNIN8 interacting with sucrose initiated the signal, and endothermic OsNIN8 interacting with OsFKBP12 transmitted the sucrose signal to stimulate OsTOR activities. The initiation and the transmission of the sucrose signal were two separate processes, though sucrose, OsNIN8, OsFKBP12, and OsTOR bound one another forming a complex or in succession.

OsNIN8 interacting with sucrose dissipated energetic strain for the initiation of sucrose signal but OsNIN8 interacting with OsFKBP12 accumulated energetic strain for transmission of sucrose signal.

So, loose associations between sucrose and OsNIN8 and among OsNIN8-OsFKBP12-OsTOR ensured this thermodynamic state for signaling but any compact associations in these interactions changed the state to block the signaling (Figure 5D).

### Limitations of the study

Loose interaction between proteins should be a manner of functional regulation of protein interaction. In fact, protein-protein interactions forming complexes performed diverse functions such as signaling^75^ and complexes could be decoded from protein-protein docking, protein-protein interface, and protein-protein interaction.^76^ Typically, hot spots, which were consist of a number of residues contributing free energy to bury in hydrophobic cavities, cause bindings between proteins in the complex.^76^ Thermodynamic and kinetic parameters of binding could be determined by many methods such as ITC, SPR (surface plasmon resonance), fluorescence spectroscopy, spectrophotometric assays, radio ligand binding, and stopped-flow fluorimetry to assess the binding affinity.^75^

Whereas, in the case of OsFKBP12 binding to OsNIN8 or to OsNIN8m the phase reversals and heat rate enlargement after titration saturation interfered value of parameters. Fortunately, their bindings could be assessed between exothermic and endothermic rather than by numerical values. So, it required comparability not only of protein molecules but also of binding thermodynamics for ITC assessment.

In addition, the loose interaction of OsNIN8 with OsFKBP12 was only displayed in the double transformation of AD and BD vectors in yeast, but not in co-immunoprecipitation (pull-down) or BiFC assays. It indicated that the sensitivity of an interaction was different in using different approaches.

Therefore, an appropriate approach for binding a pair of proteins was important in loose interaction assay. And a universal approach for this loose interaction was expected.

## Resource availability

### Lead contact

Requests for resources and reagents should be directed to the lead contact, Zizhang Wang (zizhangw@ibcas.ac.cn).

### Materials availability

This study did not generate new unique reagents.

### Data and code availability

• Data reported in this publication will be shared upon request from the lead contact.
• This study did not generate unique code.
• Any additional information required to reanalyze the data reported in this article is available from the lead contact upon request.

## Acknowledgments

The authors thank Prof. Tai Wang for experiment conditions and guidance; Jingquan Li for confocal microscopy; Zheng Fan of Institute of Microbiology, Chinese Academy of Sciences for ITC determination. This study was supported by the 10.13039/501100002367Chinese Academy of Sciences, China. (grant No. XDA08010301).

## Author contributions

Z. W. designed the research, conducted biological and thermodynamic experiments, and wrote the article. H. L. and X. W. performed Fourier transform infrared spectroscopy assay.

## Declaration of interests

The authors declare no conflicts of interest.

## STAR★Methods

### Key resources table

**Table:** 

REAGENT or RESOURCE	SOURCE	IDENTIFIER
**Antibodies**		

c-Myc epitope monoclonal antibody	Clontech	Cat# 631206; RRID: AB_2928131
Monoclonal anti-HA antibody	Sigma-Aldrich	Cat# H9658; RRID: AB_260092
Mouse anti-maltose binding protein monoclonal antibody	MBL	Cat# M091-3; RRID: AB_592157
Mouse anti-His tag monoclonal antibody	MBL	Cat# JM-3646-100; RRID: AB_591875sxz
Goat anti-mouse IgG-HRP secondary antibody	Santa Cruz	Cat# sc-2031; RRID: AB_631737
Rabbit anti-OsS6K1 polyclonal antibody	This paper	anti-OsS6K1 antibody; RRID: AB_2832919
Rabbit anti-OsS6K1^T464p^ polyclonal antibody	This paper	anti-OsS6K1^T464p^ antibody; RRID: AB_2181314

**Bacterial and virus strains**		

*E.coli* DH5α competent cell	TIANGEN	Cat# CB101
BL21(DE3) chemically competent cell	TRANSGEN	Cat# CD601

**Chemicals, peptides, and recombinant proteins**		

Tryptone	OXOID	Cat# LP0042B
Yeast extract powder	OXOID	Cat# LP0021B
D-Glucose, anhydrous	Mei5bio	Cat# G47857
Sucrose	MP Biomedicals	Cat# 04821721
D-Raffinose	Lablead	Cat# J392
Sucrose octaacetate	Macklin	Cat# D801264
Sucralose	Aladdin	Cat# S107614
Lactose	BBI	Cat# A604280-0500
Maltose	Fluka	Cat# M63420
Fructose	Sigma-Aldrich	Cat# PHR1002
Cellobiose	Macklin	Cat# D805315
Amylose Resin	Biolads	Cat# E8021
Amylose magnetic beads	Biolands	Cat# E8035
Ni-NTA His. Bind Resin	Millipore	Cat# 70666-4
YPDA	Clontech	Cat# 630306
Minimal SD Base	Clontech	Cat# 630411
DO supplement -Leu/-Trp	Clontech	Cat# 630417
DO supplement -Ade/-His/-Leu/-Trp	Clontech	Cat# 630428
RNeasy plant mini kit	Qiagen	Cat# 74903
SuperScript Ⅲ first-strand synthesis system	Invitrogen	Cat# 18080-051
Power SYBR™ Green PCR Master Mix	Applied Biosystems	Cat# 4367659
Luciferase Assay System with Reporter Lysis Buffer	Promega	Cat# E4030
N-Succinyl-Ala-Ala-Pro-Phe-P-Nitroanilide	Sigma-Aldrich	Cat# S7388
Chymotrypsin	Meridian	Cat# A50142H
Rapamycin	Biorbyt	Cat# orb61092
Torin2	Immunoway	Cat# MC0120
AZD8055	Macklin	Cat# A837230
FK506	Abcam	Cat# ab120223

**Experimental models: Organisms/strains**		

*nin8* rice mutant	This paper	*nin8*
*OsNIN8* CRISPR/Cas 9 knockout line 1	This paper	*OsNIN8* CR 1
*OsNIN8* CRISPR/Cas 9 knockout line 2	This paper	*OsNIN8* CR 2
*OsNIN8* overexpression line 6	This paper	*OsNIN8* OE 6
*OsNIN8* overexpression line 8	This paper	*OsNIN8* OE 8
*OsNIN8* RNAi knockdown line 2	This paper	*OsNIN8* Ri 2
*OsNIN8* RNAi knockdown line 6	This paper	*OsNIN8* Ri 6
*OsFKBP12* CRISPR/Cas 9 knockout line	This paper	*OsFKBP12* CRI
*OsFKBP12 CRI* 1+ *OsFKBP12 F29-Q112* complementation line	This paper	*OsFKBP12* CRI+ *OsFKBP12* F29-Q112
*OsFKBP12 CRI* 1+ *OsFKBP12 S41-Q112* complementation line	This paper	*OsFKBP12* CRI+ *OsFKBP12* S41-Q112
*OsTOR* RNAi knockdown line	This paper	*OsTOR* RNAi

**Oligonucleotides**		

CGCCGGAATGCCAGAAG	Tsingke	NIN8qF1
ATCAAACCCCTCAGACAAGCA	Tsingke	NIN8qR1
TTTGATACCTTCCCAACATTGCT	Tsingke	NIN8qF2
ACACCCATTCTGCGATCTATCAT	Tsingke	NIN8qR2
GAGGCGCTGGTGCTCAA	Tsingke	u68qF
GGCAATATTGGCGGAATCCT	Tsingke	u68qR
ACGTACGGACAGAGCCAAGAG	Tsingke	u80qF
CGTGATGTCATCGAAATTCCA	Tsingke	u80qR
GCTGTGACAAAGGCAGCAGAT	Tsingke	u86qF
TCAGGATTGCCGGTGTTGTA	Tsingke	u86qR
CCCGGTGCTCACCATGAC	Tsingke	u89qF
AAGGCGTGCCACATCGA	Tsingke	u89qR
CACAGTGCTATTCATGGTGCAA	Tsingke	u128qF
TCTGCCGTTGACATTTCCAA	Tsingke	u128qR
GCCACTCTCCACTGACCACAA	Tsingke	u133qF
CTGCTTCCACTCGTTCCAACT	Tsingke	u133qR
TTGCAGCCATGGCGAAT	Tsingke	u136qF
GGACCCCAGTCAGCTCAAGA	Tsingke	u136qR
GCCAAGGTGGATCCTGACAA	Tsingke	u154qF
CCACAGCATTCTAACTCGGAAGT	Tsingke	u154qR
TCGATATGGGCCGTTGGTT	Tsingke	u173qF
GTCATGGCGGCGATCAG	Tsingke	u173qR
TCACCGACGGCGATGAGT	Tsingke	u175qF
TGTTAAATGCCGGGTGGACTA	Tsingke	u175qR
AGGCCCGGCATGAAGAC	Tsingke	u177qF
GGCCTGAGCATGCAACCT	Tsingke	u177qR
CTCGTCACAAGCCGCTTACA	Tsingke	u200qF
GTGTTTGTGGTGGGAAATGGT	Tsingke	u200qR
CGGCCGTCCACCTACTTCT	Tsingke	u204qF
AGTACTGCAAAACCAGGGAGAGA	Tsingke	u204qR
CCTATGGCTGTACCTCGTCTTGT	Tsingke	u226qF
CGGTGCGGCAATACGAA	Tsingke	u226qR
ACGAGTTGCCATTCTGTGG	Tsingke	TORF
CTCCATCATAGCCATAACGC	Tsingke	TORR
TGAAGGTATTGGGCTTGATG	Tsingke	S6K1F
GAAGAGGGAGGCTTGACAT	Tsingke	S6K1R
TGTTAGCCAATCTTTGCCAC	Tsingke	S6K2F
CTGCTGAACTTTGTCCCTG	Tsingke	S6K2R
AGGAATGGAGAATCTGCG	Tsingke	RPA1F
AAAGTGTTGAGCGTCATCTG	Tsingke	RPA1R
TGCGGTGCTATCATACACA	Tsingke	RPA2F
TGGCAGGCAAAGTTCTTG	Tsingke	RPA2R
TGGTGGAGTATCTGGAATAGC	Tsingke	CBE1F
GGTGATTGCCTCTCAGTTCT	Tsingke	CBE1R
AACTGGTATCGTGTTGGACTC	Tsingke	ActinF
GTATTTCCTCTCAGGCGGT	Tsingke	ActinR

**Recombinant DNA**		

Plasmid: pGBKT7:*OsNIN8*	This paper	*BD-OsNIN8*
Plasmid: pGBKT7:*OsNIN8m*	This paper	*BD-OsNIN8m*
Plasmid: pGBKT7:*OsTORKD*	This paper	*BD-OsTORKD*
Plasmid: pGBKT7:*OsTORKD-FRB*	This paper	*BD-OsTORKD-FRB*
Plasmid: pGBKT7:*OsTORFRB*	This paper	*BD-OsTORFRB*
Plasmid: pGADT7 AD::*OsFKBP12*	This paper	*AD-OsFKBP12*
Plasmid: pGADT7 AD::*OsFKBP12F29-Q112*	This paper	*AD-OsFKBP12F29-Q112*
Plasmid: pGADT7 AD::*OsFKBP12S41-Q112*	This paper	*AD-OsFKBP12S41-Q112*
Plasmid: pETMALc-H::*OsNIN8*	This paper	*MBP-OsNIN8*
Plasmid: pETMALc-H::*OsNIN8m*	This paper	*MBP-OsNIN8m*
Plasmid: pETMALc-H::*OsTORKD*	This paper	*MBP-OsTORKD*
Plasmid: pETMALc-H::*OsTORKD-FRB*	This paper	*MBP-OsTORKD-FRB*
Plasmid: pETMALc-H::*OsTORFRB*	This paper	*MBP-OsTORFRB*
Plasmid: pET28a:*OsFKBP12*	This paper	*His-OsFKBP12*
Plasmid: pCAMBIA 1390:*OsFKBP12F29-Q112*	This paper	*OsFKBP12F29-Q112* comp.
Plasmid: pCAMBIA 1390:*OsFKBP12S41-Q112*	This paper	*OsFKBP12S41-Q112* comp.
Plasmid: pTCK303:*OsNIN8^7^*^*89−*^*^1050^*^*bp*^ RNAi	This paper	*OsNIN8* RNAi
Plasmid: pTCK303:*OsTOR*^*5686-6042bp*^ RNAi	This paper	*OsTOR* RNAi
Plasmid: pCAMBIA 1391:*ProUBI*-*OsNIN8*	This paper	*OsNIN8* OE

**Software and algorithms**		

HISAT software	Hopkins	Alignment
HTseq software	python	Location and count
DEGseq	Tsinghua	Differential analysis
DESeq	Bioconductor	Differential analysis
GOseq	R project	Enrichment
DNAMAN v6	Lynnon	Alignment
StepOnePlus system	Applied Biosystems	Real-time PCR
NanoAnalyze v3.7.5	TA Instruments	ITC
Origin 93	Northampton	FTIR
Excel software	Microsoft	Statistical analysis
ImageJ 1.46	NIH	Quantification
ProtScale	https://web.expasy.org/protscale/	Hydrophobicity

### Experimental model and study participant details

#### Rice materials and growth conditions

Sucrose treatment of rice sprouts. Rice unshell seeds were soaked in water for 8 h and transferred on wet filter paper in a culture dish keeping at 24°C in dark for 4 days, when they were as early as possible for sample collection during germination. Sprout including shoot, plumular axis and radicle were removed from endosperm tissue along the scutellum carefully to keep sprouts undamaged. Debris of endosperm were washing-out three times in water. Sprouts fasted on wet paper for another 5 days to run out of sugars. Sucrose used for treatment was 175 mM, which was the best concentration for inducting callus in rice,^77^ Sprouts were soaked in sucrose aqueous solution for 1 h or 6 h with gently shake for aeration. The culture and treatment were performed in the culture room to keep temperature unchanged and kept in dark as much as possible.

Treatment of TOR-specific chemical inhibitors on seeds during germination. Four inhibitors, AZD8055 (Macklin, A837230), Torin 2 (Immunoway, MC0120), Rapamycin (Biorbyt, orb61092) and FK506 (abcam, ab120223), all were hydrophobic and required to dissolve in dimethyl sulfoxide (DMSO), but concentration of DMSO in the medium greater than 10 μL/mL would inhibit growth of sprout during germination in rice (Figure S8). To optimize effective concentration of inhibitors but avoid effects of inhibit on growth from DMSO for the treatment, exposure concentrations of AZD8055, Torin2, rapamycin and FK506 were 300, 85, 480 and 45 μM in 1/2 MS solid medium, respectively, after concentration gradient experiments (Figure S8). Seeds were inoculated in test tube or culture flask and cultured at 24°C with 12 h light and 12 h dark cycle for 7 days.

Knockout of *OsFKBP12* and its complementation with 3′ fragments of *OsFKBP12*. Knockout of full length *OsFKBP12* was using CRISPR/Cas9 approach in ZH11 by Biogle company (Hangzhou, China). *Cas9* gene in knock out lines were eliminated by backcross with ZH11. Genotypes of segregant were confirmed by PCR and sequencing. The 85–339 bp region (encoded 29–112 AA) or the 121–339 bp region (encoded 41–112 AA) of *OsFKBP12* CDS were added ATG to 5′-end by primers and sub-cloned under UBI promoter in pCAMBIA 1391 vector mediated by PCR, and transformed into *OsFKBP12* knockout line (*OsFKBP12*^*CRI*^) for replace the full-length with 3′ fragments of OsFKBP12.

Knockout of *OsTOR* and *OsTOR* RNAi. An attempt of knockout *OsTOR* were performed using CRISPR/Cas9 method but all regenerated seedlings were not biallelic inactivation, and phenotype of these heterozygotes were not changed from ZH11 including in radicle and seed setting. Target sequence for RNAi was the FRB region (5686-6042bp of CDS) of *OsTOR*, this fragment was forward and reverse inserted into pTCK303 and was transformed into ZH11.

### Method details

#### RNA-seq and real-time PCR

Total RNAs were extracted to perform RNA-seq analysis. RNA isolation was using RNeasy plant mini kit (Qiagen, 74903). Experiment was designed with three biological replicates.

Sample quality control, RNA-Seq and quantification analyses were conducted by Total Genomics Solution company (Shen Zhen, China) following their experiment workflow. Real-time PCR was performed using StepOnePlus system (Applied Biosystems), PCR primers were designed by Primer Express v3.0 tool of the system.

#### Luciferase assay

Promoter fragments of u80, u128, u133 and u173, which were 2001, 2186, 2012 and 1982 bp in length, respectively, were cloned from genomic DNA of ZH11 and subcloned into reporter vector^78^ for driving expression of luciferase.

Protoplast preparation and transient expression were carried out as described previously.^79^ In brief, false stems of etiolated ZH11 seedling at 10 DAI, which were removal of seeds, roots and blades, were basal ends soaked in 0.6 M D-mannitol, 10 mM MES, pH 5.8 for 24 h in darkness for starvation of sugars. Protoplasts of 1x10^6^/mL were prepared from these stems and aliquot of 100 μL was transiently transformed with 10 μg reporter vectors, simultaneously transformed with 6 μg *35S::GUS* each as the normalization control as described.^78^ Transformants were incubated at 25°C in darkness for 15 h and treated with sucrose of 10, 20 and 100 mM or glucose of 10 and 100 mM for 3 h.

Luciferase activities were conducted using luciferase assay system (Promega, E4030 and E1531). GUS activities were conducted using 4-methylumbellifery β-D-glucuronic acid as substrate and reactions were stopped with Na_2_CO_3_ solution as described.^78^ Fluorescences were detected by Glomax detector (Promega).

#### Yeast two-hybrid (Y2H) assay

Roots of ZH11 at 4 DAI were collected and total RNA was isolated and transcribed reversely into cDNA for construction of a two-hybrid library using Mate & Plate library system (Clontech, PT4085-1). *OsNIN8* was subcloned into pGBKT7 for fusion with GAL4 DNA-BD as a bait and the plasmid was transformed into Y2H Gold strain. Yeast mating between the library strain and the bait strain for library screening using Matchmaker gold yeast two-hybrid system (Clontech, PT4084-1).

#### Construction of vectors for OsFKBP12 or its C-terminal fragments fusing to GLA4 AD

Two regions of 85–339 bp and 121–339 bp of *OsFKBP12*, both of which were added ATG to 5′-end mediated by PCR primers, as well as the full length *OsFKBP12* (1–339 bp) were sub-cloned into MCS of pGADT7 AD vector of the Y2H system.

#### Construction of vectors for fusion with GLA4 BD

Following the construction of bait vector, encoding regions of KD (H1896-W2465), FRB domain (H1896-T2014) and KD without FRB (KD-FRB, T2015-W2465) of OsTOR fragments, all of which were added ATG to 5′-end mediated by PCR primers, as well as CDS of *OsNIN8m* were sub-cloned into pGBKT7 vector of the Y2H system.

#### Protein purification of OsTORKD and OsFKBP12

*OsNIN8*, *OsNIN8m* and *OsTORKD* (GenBank: Os05g0235300, fragment 5686-7398bp of *OsTOR* CDS) were cloned into pETMALc-H to generate MBP-OsNIN8, MBP-OsNIN8m and MBP-OsTORKD, respectively. *OsFKBP12* (RAP-DB: LOC_Os02g52290) and Os14-3-3 (RAP-DB: LOC_Os02g36974) were cloned into pET 28a (+) (Novagen) to generate His-OsFKBP12 and His-Os14-3-3, respectively. Proteins were expressed in *E.coli* BL21 (DE3) (Biolabs) with 0.1 mM IPTG at 25°C for 4 h. Proteins with MBP tag were purified using Amylose Resin (Biolads, E8021). Proteins with His tag were purified using Ni-NTA His. Bind Resin (Millipore). Proteins were dialyzed using 12–14 kD dialysis membrane (Spec tra/Por) in 50 mM potassium phosphate buffer (pH6.4). Protein concentrations were determined by Braford reagent (Sangon, Shanghai, China).

#### Pulldown assay

Pulldown assay of protein interactions were performed as described previously.^79^ Tags for protein purification and immunoassay were as indications.

#### Bimolecular fluorescence complementation (BiFC) assay

BiFC assay was followed the description using pSPYNE173 and pSPYCE(M), plasmids were co-transformed transiently into mesophyll cells of tobacco mediated by EHA105 strain.^80^ Imaging was visualized using Leica TCS SP5 confocal laser scanning microscopy.

#### ITC titration assay

ITC assay was using Affinity ITC LV (TA Instruments, New Castle, DE, USA) with NanoAnalyze software v3.7.5 (TA Instruments). Protein concentration in the sample cell was 0.1 mM, that in the syringe were 10 mM, temperature was 25°C. The titration was: injection interval 200 s, volume 2.5 μL for 20 injections.

#### Peptidyl-prolyl *cis*-*trans* isomerase activity of OsFKBP12

*Cis*-*trans* isomerization on the X-Pro peptide bond of N-Succinyl-Ala-Ala-Pro-Phe-P-Nitroanilide (Sigma S7388) by OsFKBP12 was performed as described.^35^ The reaction was set including substrate 20 μM, chymotrypsin (Meridian, A50142H) 2 μg and OsFKBP12 25 μg in 100 μL 50 mM potassium phosphate buffer (pH 6.4) and recorded the OD_395_ for 2 min with 20 s interval.

#### FTIR spectroscopy assay

Proteins were performed concentrating and reducing salt ions and replaced the potassium phosphate buffer with D_2_O buffer containing 50 mM K_2_DPO_4_/KD_2_PO_4_ (pD 6.4) for five times. Concentrations of MBP-OsTORKD were adjusted to 0.1 mM and His-OsFKBP12 to 0.6 mM. FTIR spectra were collected on a spectrometer (ABB-BOMEM, Bureau, Canada) equipped with a liquid nitrogen cooled broad band mercury-cadmium-telluride detector.

To compare OsTORKD with (OsTORKD+OsFKBP12) spectra for removing the interference of MBP and His tags. Four determinations were performed and collected datasets of spectrum, respectively: (a) MBP-OsTORKD, (b) (MBP-OsTORKD)+(His-OsFKBP12), (c) MBP and (d) MBP+(His-FKBP12). Spectrum of OsTORKD = (a)-(c), and that of (OsTORKD+OsFKBP12) = (b)-(d). Data analysis was performed using Origin 93 program (Northampton, MA, USA) as previous description^81^ for second derivative intensity.

#### Preparation of phospho-OsS6K1T^464^ polyclonal antibody and western blotting

A 15-AA peptide AGGAGHSSFAGFT^464^YV of OsS6K1 (RAP-DB: LOC_Os07g48290) was designed as antigen sequence. Peptide synthesis, phosphorylation modification, immunization of rabbits and antisera affinity purification were performed by Abcam (Shanghai, China). Protoplast of ZH11 and *nin8* were prepared from false stems of sucrose starvation treatment. Aliquot of 800 μL with 2x10^5^/mL were transiently transformed with 80 μg pUBI::S6K1 and incubation for 16 h and then added sucrose to final 20 mM for 4 h. Cells were harvested by centrifugation, lysed in RIPA buffer treating with ultrasonic 3 s for 20 times, the centrifuged supernatant was suspended in loading buffer followed the protocol of Abcam (www.abcam.com/technical). Western blotting of phosphor-proteins was according to Abcam protocol. Phosphorylation antibody (2 μL of 0.22 mg/mL diluted to 200 μL) was blocked with antigen polypeptide of non-phosphorylation antibody (5 μL of 1 mg/mL) overnight and centrifugated to collect supernatant before use in western blotting to screen possible non-phosphorylation antibody.

#### RT-PCR assay of genes downstream of OsTOR responding to sucrose

Endosperm-removed ZH11, *nin8* and *nin8*+*OsNIN8D275AE501A* sprouts were treated with sucrose and total RNAs were prepared (Qiagen, 74903) for templates of RT-PCR using SuperScript Ⅲ system (Invirtrogen, 18080-051) and Q5 DNA polymerase (BioLabs M0491). Primer sequences of *OsTOR*, *OsS6K1*, *OsS6K2* (RAP-DB: LOC_Os03g21620), *OsCBE1* (RAP-DB: LOC_Os06g01680), *OsRPA1* (RAP-DB: LOC_Os06g40950), *OsRPA2* (RAP-DB: LOC_Os10g35290) and OsActin (RAP-DB: LOC_Os11g06390) were listed in Table S3. The reaction was carried out for 33 cycles of 58°C annealing.

#### Knockouts of *OsFKBP12* and *OsTOR*, *OsTOR* RNAi and *OsFKBP12* complementation

Knockouts of *OsFKBP12* and *OsTOR* were using CRISPR/Cas9 approach in ZH11. Sequence used for *OsTOR* RNAi was the FRB region (5686-6042bp of its CDS). The 85–339 bp or the 121–339 bp sequences of *OsFKBP12* CDS were 5′-end added ATG and sub-cloned under UBI promoter in pCAMBIA 1391 vector mediated by PCR, and transformed into *OsFKBP12* knockout line for replace the full-length with 3′ fragments of OsFKBP12.

#### TOR-specific chemical inhibitors treatment of rice seed

AZD8055 (Macklin, A837230), Torin 2 (Immunoway, MC0120), Rapamycin (Biorbyt, orb61092) and FK506 (abcam, ab120223) dissolved completely in DMSO to 40, 11.5, 65 and 6 mM, respectively. These stocks and DMSO were added into 1/2 MS solid medium with different concentrations to culture aseptically rice glume-removed seeds for determining their effectiveness and DMSO disturbance.

### Quantification and statistical analysis

#### Quantification and statistical analysis of RNA-seq

The reference genome was Oryza sativa japonica group (NCBI ID 39947). Alignment and location of clean reads to exon region of chromosomes were using HISAT and HTseq softwares, respectively. Calculation of gene expression was using FPKM method. Software for differential analysis was using DEGseq and DESeq with log2Fold >2 and q-value <0.01. GO enrichment analysis was using GOseq software with *p*-value <0.05.

#### Selection of DEGs for screening report genes from DEGs of RNA-seq

From the DEGs of treatment of sucrose for 1 h against without treatment (3,637 DEGs including 1,929 up-regulation and 1,708 down-regulation), firstly the 200 highest DEGs from sucrose treatment were selected, then the 200 lowest DEGs from the control (treatment with H_2_O) were selected, additionally, the 200 greatest change-fold DEGs between treatment with and without sucrose were selected. Totally, an integration of three selections were 335 genes including 253 up-regulation and 82 down-regulation genes. These selections were not related to gene function, but only to consider of sufficient difference for real-time PCR in follow-up assays.

#### Quantification of blotting band

Software for quantifying blotting band was using ImageJ 1.46 (National Institutes of Health, USA).

Quantifications of FTIR spectroscopy, ITC enthalpy change, and quantitative PCR were detailed in the method details section. Experimental replication was indicated in the Figure legends and in the method details section.

The statistical analysis was implemented using Microsoft Excel software for student’s-test, and statistical difference was assigned when *p* values were <0.05 and significant difference when *p* values were <0.01.

## Supplemental information

Document S1. Figures S1–S8 and Table S3

Table S1. Differential expression and enrichment analyses of RNA_Seq, related to Figure 1

Table S2. DEGs selected for screening report genes, related to Figure 2, Tables 1 and 2
