# Supplementary material for: OsFKBP12 transduces the sucrose signal from OsNIN8 to the OsTOR pathway in a loosely binding manner for cell division
Source: iScience. 2024 Dec 9;28(1):111555. doi: 10.1016/j.isci.2024.111555 (PMC11732086; doi:10.1016/j.isci.2024.111555)
Supplement: Document S1. Figures S1–S8 and Table S3 [file mmc1.pdf]

iScience, Volume *28*

Supplemental information

OsFKBP12 transduces the sucrose signal from OsNIN8 to the OsTOR pathway in a loosely binding manner for cell division

Zizhang Wang, Hao Li, and Yuxiang Weng

	RAF		SOA		SCL		CBO		SUC		LAC		MAL		GLU		FRU		SUM
	+	−	+	−	+	−	+	−	+	−	+	−	+	−	+	−	+	−	
U68	5	1	5	1	4	2	6	0	6	0	5	1	5	1	6	0	3	3	7
U80	3	3	4	2	4	2	4	2	6	0	6	0	5	1	3	3	0	6	3
U86	4	2	5	1	4	2	5	1	5	1	5	1	5	1	4	2	4	2	5
U89	5	1	5	1	5	1	4	2	5	1	4	2	5	1	3	3	3	3	5
U128	5	1	6	0	6	0	6	0	6	0	5	1	6	0	5	1	5	1	9
U133	5	1	5	1	6	0	6	0	6	0	6	0	5	1	4	2	2	4	7
U136	3	3	6	0	6	0	5	1	5	1	6	0	5	1	4	2	5	1	7
U154	2	4	3	3	3	3	2	4	5	1	4	2	1	5	2	4	2	4	1
U173	4	2	6	0	5	1	5	1	6	0	6	0	6	0	5	1	3	3	7
U175	4	2	6	0	4	2	4	2	5	1	5	1	5	1	3	3	5	1	5
U177	6	0	6	0	5	1	5	1	5	1	4	2	5	1	5	1	3	3	7
U200	3	3	5	1	5	1	5	1	6	0	2	4	5	1	4	2	3	3	5
U204	2	4	5	1	4	2	4	2	5	1	5	1	4	2	1	5	0	6	3
U226	5	1	4	2	5	1	5	1	5	1	3	3	5	1	4	2	4	2	5
SUM	6		11		8		9		14		9		12		4		3		

Figure S1. Sucrose responding genes were more sensitive to sucrose than sucrose derivatives, sucrose analogues and monosaccharide (Related to Figure 2)

Six independent experiments were conducted for differential expressions between ZH11 and *nin8* starved sprouts after treatment with different reagents using real-time PCR. Gene expression being significant greater in ZH11 than *nin8* (n=3, t<0.05) was assigned as positive (+), otherwise, as negative (-). Positive results from five and six experiments as stably positive responses (underlined in red), otherwise, as unstable positive (not underlined). Row sum, times of stably positive responses among reagents in total; Column sum, times of stably positive responses among genes in total. RAF, raffinose; SOA, sucrose octaacetate; SCL, sucralose; CBO, cellobiose; SUC, sucrose; LAC, lactose; MAL, maltose; GLU, glucose; FRU, fructose

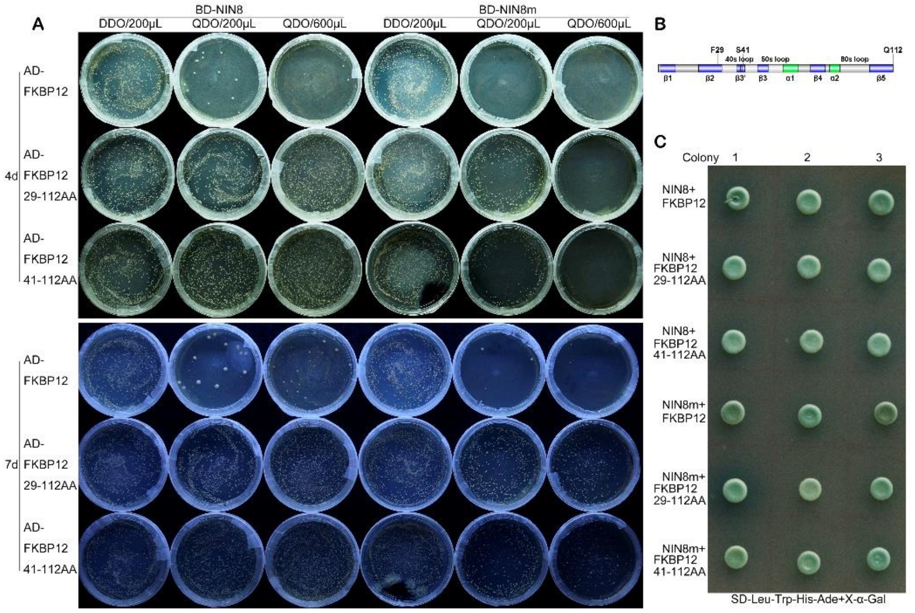

Figure S2. Validation of full-length (M1-Q112), F29-Q112 and S41-Q112 fragments of OsFKBP12 association with OsNIN8 or OsNIN8m each other in Y2H (Related to Figure 3)

(A) Plate cultivations of co-transformation with different AD and BD vectors, 200 μL for normal spread and 600 μL for heavy spread, and cultured for 4 d and 7 d. DDO, growth medium; QDO, selection medium for interaction. AD-OsFKBP12 with OsNIN8 or OsNIN8m shown the

loose fashion of associations. Growths of two OsFKBP12 fragments with OsNIN8 were faster than those with OsNIN8m (column 3 vs column 6).

(B) Schematic of secondary structure of OsFKBP12. OsFKBP12 were searched for protein structure on SWISS-MODEL, it matched the *Aspergillus fumigatus* FKBP12 at the highest GMQE of 0.78. Similar to other FKBP12, it consisted of β1-β2-40s loop-β3-50s loop-α1-β4-80s loop-β5. F29 and S41 on OsFKBP12 were indicated.

(C) Regrowth of colonies with X-α-Gal added on QDO medium.

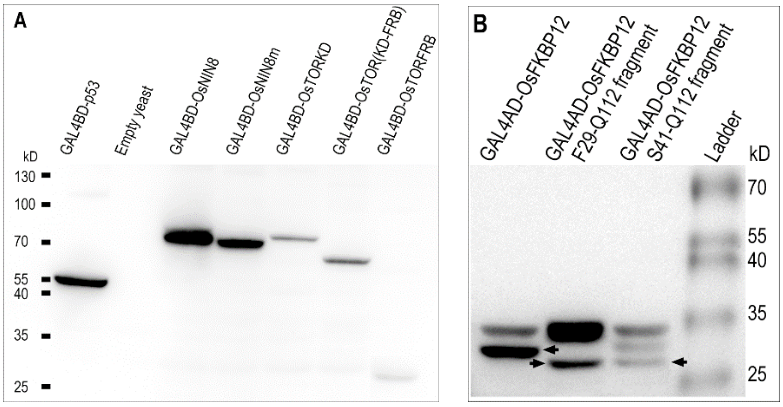

Figure S3. Expression of international proteins in yeast during Y2H (Related to Figures 3 and 4)

(A) Expression of proteins fused with GAL4 DNA BD were detected with c-Myc epitope antibody.

(B) Expression of proteins fused with GAL4 AD were detected with HA-tag antibody.

These genes were constructed in-frame into vectors pGBKT7 or pGADT7 AD of the Y2H system, these vectors were transformed into yeast cell, yeast proteins were extracted and subjected to immunoblotting analyses. Ladder was pre-stained proteins and was merged to images of immunoblotting. Target bands were indicated in (B).

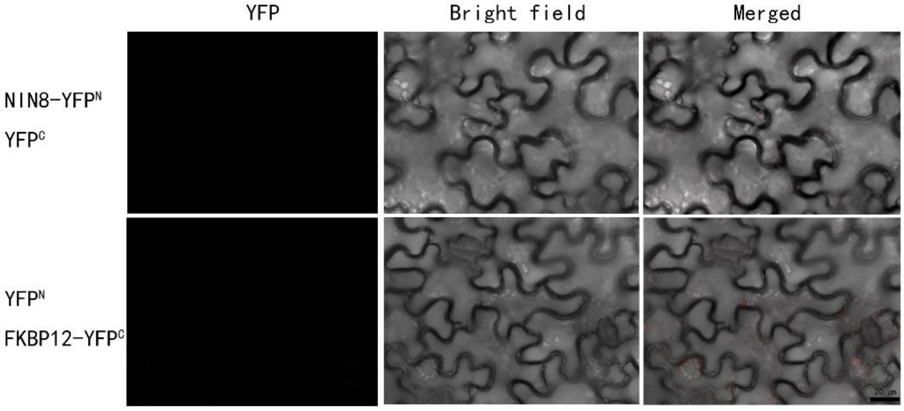

Figure S4. OsNIN-YFP^{N} and YFP^{C} or YFP^{N} and OsFKBP12-YFP^{C} did not interact (Related to Figure 4)

They did not pull together to emit fluorescence in the mesophyll cells serving as negative controls.

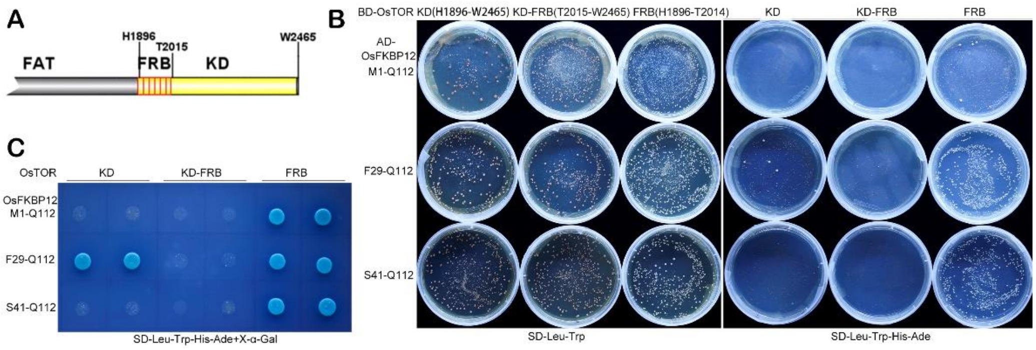

Figure S5. Validation of full-length (M1-Q112), F29-Q112 and S41-Q112 fragments of OsFKBP12 association with TORKD (H1896-W2465), KD-FRB (T2015-W2465) or FRB (H1896-T2015) each other in Y2H (Related to Figure 4)

(A) Schematic of conserved domains of OsTOR. FRB region harbored in the front of KD region.

(B) Plate cultivations of co-transformation with different AD and BD vectors on DDO (SD-Leu-Trp) or QDO (SD-Leu-Trp-His-Ade) media. Growths of OsFKBP12M1-Q112 with OsTORFRB or OsFKBP12F29-Q112 with OsTORKD shown the loose fashion of association.

(C) Regrowth of colonies with X-α-Gal added on QDO medium

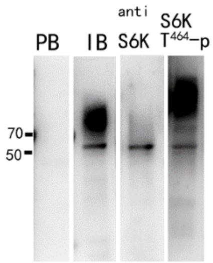

Figure S6. Preparation of non-phosphorylation (anti-S6K) and phosphorylation (anti-S6K T^{464}-p) antibodies (Related to Figure 4)

Protein sample is prepared from protoplast of ZH11 transiently transformed with S6K1 and treated with sucrose. Phosphorylation antibody is blocked with antigen polypeptide of non-phosphorylation to screen possible non-phosphorylation antibody before use. PB, pre-immune serum; IB, post-immune serum; anti-S6K, expression of S6K; anti-S6K $T^{464\text{-p}}$, phosphorylation level of S6K $T^{464\text{-p}}$.

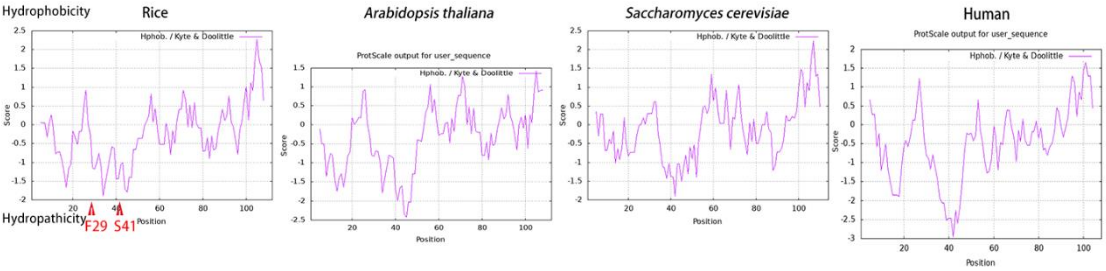

Figure S7. Hydrophobicity analysis of OsFKBP12s from different species (Related to Figure 4)

Amino acid sequence was submitted to ProtScale (https://web.expasy.org/protscale/) for

hydrophobicity analysis. Datum lines and vertical axis scales had been aligned. Score>0, hydrophobicity; Score<0, hydropathicity; Truncated positions of fragment 1 and 2 at F29 and S41 are indicated in red arrowhead.

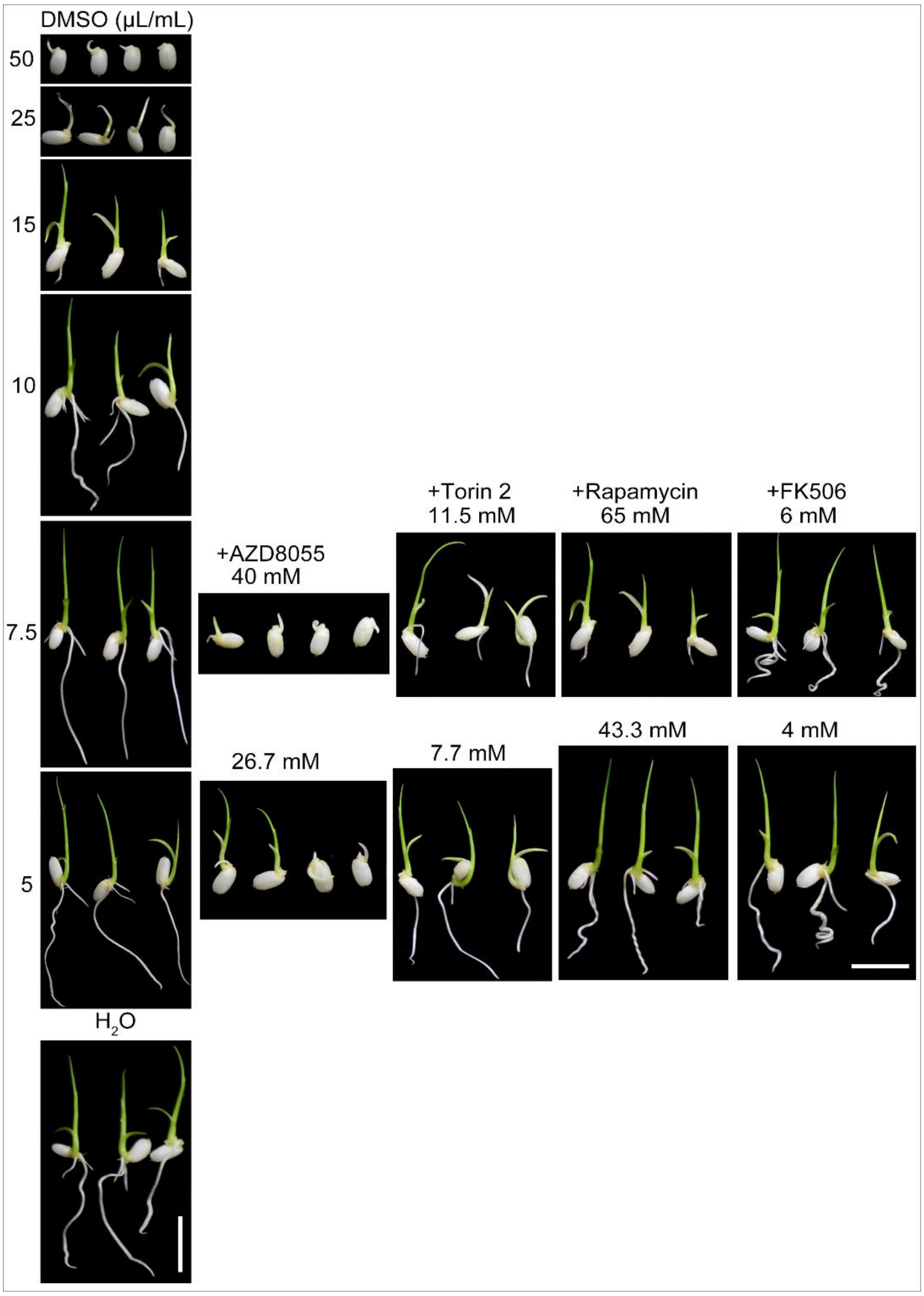

Figure S8. Determination of exposure concentration of TOR-specific inhibitors (Related to Figure 5)

High concentration of DMSO, the solvent of hydrophobic inhibitors, inhibited growth of rice sprout (column 1), concentration of DMSO in media was determined at 7.5 μL/mL, and the inhibitors were required of completely dissolved in the media. Concentrations used in the treatment of AZD8055, Torin 2, Rapamycin and FK506 were 40, 11.5, 65 and 6 mM, respectively, after concentration gradient assay. Bar=1 cm.

Table S3. Primers of RT-PCR of genes downstream of OsTOR (Related to Figure 4)

Primer	Sequence
TORF	ACGAGTTGCCATTCTGTGG
TORR	CTCCATCATAGCCATAACGC
S6K1F	TGAAGGTATTGGGCTTGATG
S6K1R	GAAGAGGGAGGCTTGACAT
S6K2F	TGTTAGCCAATCTTTGCCAC
S6K2R	CTGCTGAACTTTGTCCCTG
RPA1F	AGGAATGGAGAATCTGCG
RPA1R	AAAGTGTTGAGCGTCATCTG
RPA2F	TGCGGTGCTATCATACACA
RPA2R	TGGCAGGCAAAGTTCTTG
CBE1F	TGGTGGAGTATCTGGAATAGC
CBE1R	GGTGATTGCCTCTCAGTTCT
ActinF	AACTGGTATCGTGTTGGACTC
ActinR	GTATTTCCTCTCAGGCGGT
